# Pyrenosetins A–C, New Decalinoylspirotetramic Acid Derivatives Isolated by Bioactivity-Based Molecular Networking from the Seaweed-Derived Fungus *Pyrenochaetopsis* sp. FVE-001

**DOI:** 10.3390/md18010047

**Published:** 2020-01-11

**Authors:** Bicheng Fan, Pradeep Dewapriya, Fengjie Li, Martina Blümel, Deniz Tasdemir

**Affiliations:** 1 GEOMAR Centre for Marine Biotechnology (GEOMAR-Biotech), Research Unit Marine Natural Products Chemistry, GEOMAR Helmholtz Centre for Ocean Research Kiel, Am Kiel-Kanal 44, 24106 Kiel, Germany; bfan@geomar.de (B.F.); pdewapriya@geomar.de (P.D.); fli@geomar.de (F.L.); mbluemel@geomar.de (M.B.); 2 Faculty of Mathematics and Natural Sciences, Kiel University, Christian-Albrechts-Platz 4, 24118 Kiel, Germany

**Keywords:** marine fungus, pyrenosetin, phomasetin, *Pyrenochaetopsis* sp., *Fucus vesiculosus*, bioactivity-based molecular networking, decalin tetramic acid

## Abstract

Marine algae represent a prolific source of filamentous fungi for bioprospecting. In continuation of our search for new anticancer leads from fungi derived from the brown alga *Fucus vesiculosus*, an endophytic *Pyrenochaetopsis* sp. FVE-001 was selected for an in-depth chemical analysis. The crude fungal extract inhibited several cancer cell lines in vitro, and the highest anticancer activity was tracked to its CHCl_3_–soluble portion. A bioactivity-based molecular networking approach was applied to C18-SPE fractions of the CHCl_3_ subextract to predict the bioactivity scores of metabolites in the fractions and to aid targeted purification of anticancer metabolites. This approach led to a rapid isolation of three new decalinoylspirotetramic acid derivatives, pyrenosetins A–C (**1**–**3**) and the known decalin tetramic acid phomasetin (**4**). The structures of the compounds were elucidated by extensive NMR, HR-ESIMS, FT-IR spectroscopy, [α]_D_ and Mosher’s ester method. Compounds **1** and **2** showed high anticancer activity against malignant melanoma cell line A-375 (IC_50_ values 2.8 and 6.3 μM, respectively), in line with the bioactivity predictions. This is the first study focusing on secondary metabolites of a marine-derived *Pyrenochaetopsis* sp. and the second investigation performed on the member of the genus *Pyrenochaetopsis*.

## 1. Introduction

Macroalgae (seaweeds) are regarded as holobionts due to their complex epiphytic and endophytic microbiota [1]. Seaweed-derived fungi are emerging as a promising source of novel bioactive secondary metabolites for marine bioprospecting. For example, plinabulin, the synthetic *tert*-butyl analog of diketopiperazine halimide, which derives from the seaweed-derived fungus *Aspergillus* sp. [2] is currently undergoing phase III clinical trials for treatment of non-small cell lung cancer [2,3]. Over the past decades, fungi associated with brown algal genus *Fucus* have gained attention as an untapped source of fungal biodiversity [4,5]. A previous study by Flewelling et al. showed *Fucus*-associated fungi to produce antimicrobial compounds [6]. Further studies have shown that *Fucus*-derived fungi produced secondary metabolites belonging to diverse structural classes and exhibited further bioactivities. For example, the culture broth of *Fucus spiralis*-derived fungus *Phoma* sp. yielded the polyketide 5-hydroxyramulosin and 7-methoxycoumarin, which showed anticancer, antifungal and anti-HIV activities [7,8]. Another study by Lateff et al. (2003) reported a new, antioxidant isobenzofuranone derivative from *Epicoccum* sp. associated with *Fucus vesiculosus* [9]. However, a systematic research exploring bioactive metabolites from fungi associated with *Fucus* sp. is still missing.

Mass spectrometry-based molecular networking (MN) in conjunction with the publicly available web-platform Global Natural Products Social Molecular Network (GNPS) serves as an automated tool for mining large volumes of mass spectra. MN uses an untargeted metabolomics approach that powerfully processes the tandem mass spectrometry (MS/MS) fragmentation data. It is a vector-based workflow that calculates cosine scores (between 0 and 1) to determine the degree of similarity between the MS^2^ fragments. These fragment ions (nodes) will then be organized into relational networks depending on their similarity [10]. MN has been employed for rapid and successful dereplication of known compounds from complex natural extracts [11,12]. Another advantage of MN is the possibility for incorporation of additional information, such as the bioactivity data, over the network. The bioactivity mapping or bioactivity-based MN have been effectively applied in natural product research on both crude extracts and fractions obtained therefrom [13,14]. In the latter, a further bioinformatic program is employed to predict the bioactivity score of molecules according to their relative abundance in the fractions. Bioactivity-based MN (B-B MN) approach, hence assists rapid prioritization and targeted isolation of bioactive compounds, thereby accelerating natural product biodiscovery efforts.

*Fucus vesiculosus* is a habitat forming brown alga commonly found in the shallow coastal regions of Europe. In a recent study, we profiled the surface microbiome and metabolome of the Baltic *F. vesiculosus* and identified primary and secondary metabolites, including many fungal metabolites from its surface and inner tissues by massive MN coupled with DESI-imaging mass spectrometry [15]. We also reported the isolation and identification of epiphytic and endophytic fungal communities associated with *F. vesiculosus,* and applied an OSMAC approach to assess the impact of culture conditions on chemical space and anticancer potential of these filamentous fungi [12]. A fungal strain belonging to the order Pleosporales showed anticancer activity with lower toxicity to non-cancerous cells when cultivated in liquid potato dextrose medium (PDM) [12]. In the continuation of this project, we have now identified this fungus as a *Pyrenochaetopsis* sp. (strain FVE-001) by building a phylogenetic tree and comparing relationship with closely related fungal species. We further focused on isolation and characterization of its anticancer constituents. For this aim, we applied a B-B MN workflow [14] on the C18-SPE fractions obtained from the CHCl_3_ subextract of the fungus for prioritization of the active fractions and targeted isolation of new bioactive compounds. This approach enabled rapid identification of three new and one known decalinoyl tetramic acid derivatives, 1–4. Herein we outline the isolation, structure elucidation and anticancer activities of the compounds 1–4.

## 2. Results

### 2.1. Strain Isolation and Identification

**Figure 1:**
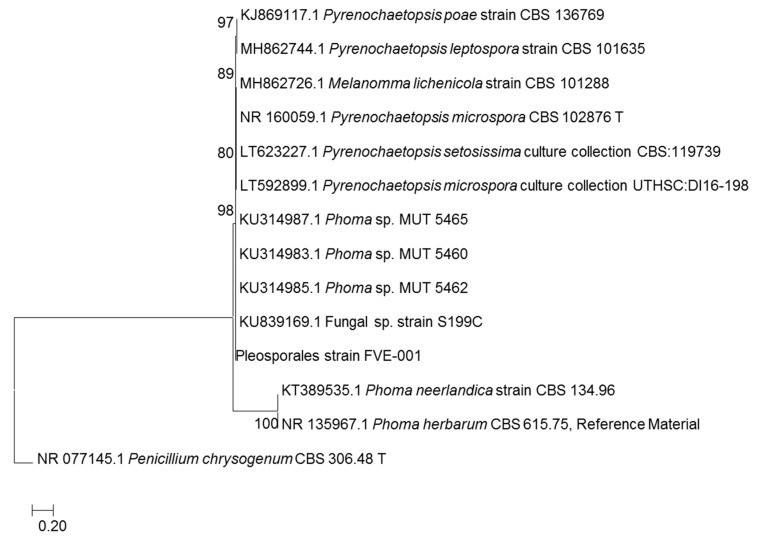
Molecular phylogenetic analysis by maximum likelihood method.

The endophytic fungus FVE-001 was isolated from the thallus of *Fucus vesiculosus* collected at Kiel Fjord (Baltic Sea, Germany) [12]. The initial Sanger sequencing of the PCR-amplified ITS1-5.8S rRNA gene-ITS2 region yielded a total length of 297 bp fragment, which only enabled its identification at order level, i.e., Pleosporales [12]. In order to further confirm the taxonomic identity of the fungus, the same genomic DNA extract was re-amplified and sequenced for ITS1-5.8S-ITS2 genes to yield a 394 bp length PCR fragment. The sequence result was subjected to NCBI Blast analysis that showed 100% sequence similarity to *Phoma* sp. and 99% sequence similarity to closely related strain, *Pyrenochaetopsis microspore*. In order to further validate the taxonomy of the fungus FVE-001, a phylogenetic tree was constructed with 14 related strains from the NCBI database. As shown in Figure 1, the phylogenetic tree suggested the fungus FVE-001 to be closely related to *Phoma* sp. However, in the phylogenetic tree, FVE-001 did not cluster with the typical *Phoma* sp., i.e., *Phoma neerlandica* and *Phoma herbarum*. Further investigation revealed that the closely related *Phoma* strains MUT 5460, MUT 5462 and MUT 5465 (Figure 1) have now been reclassified as *Pyrenochaetopsis leptospora* in the UNITE database (https://unite.ut.ee/sh/SH1525086.08FU#fndtn-panel1) [16]. This confirmed that the fungus FVE-001 to be a *Pyrenochaetopsis* sp.

### 2.2. Cultivation, Extraction, Bioactivity Test and Molecular Networking

Based on the results of our One-Strain-Many-Compounds (OSMAC) study [12], the fungus *Pyrenochaetopsis* sp. FVE-001 was cultivated at 22 °C for 14 days under shaking (120 rpm) in 2 L flasks, each containing 800 mL PDM liquid medium. The EtOAc extract of the culture broth (12 L in total) was evaporated, and subjected to a modified Kupchan partition to yield *n*-hexane (KH), CHCl_3_ (KC) and aqueous MeOH (KM) subextracts. All three subextracts were tested against five human cancer cell lines (malignant melanoma A-375; lung carcinoma A-549; colorectal adenocarcinoma HT-29; colorectal carcinoma HCT-116 and breast cancer MB-231), plus against non-cancerous human keratinocyte cell HaCaT for assessing their cytotoxicity. The KC subextract showed the highest activity against all cancer cell lines (>75% cell growth inhibition at 100 µg/mL) (Table S1), and was selected for in-depth chemical investigations. It was subjected to solid phase extraction (SPE) on a C18 Sep-Pak cartridge (10% gradient elution from 0% to 100% methanol) to yield 11 fractions. Fractions 5–7 exhibited a promising activity with high rates of growth inhibition (up to 99% at 100 µg/mL, Table S1). The fraction 8 showed a moderate inhibition rate (43%) against malignant melanoma cell line A-375 while the rest of the fractions had no activity (Table S1). In order to investigate their chemical profiles, all 11 fractions were analyzed by tandem UPLC-QToF-MS/MS (positive ion mode) metabolomics using MN. To facilitate the bioassay-guided isolation of anticancer metabolites, we integrated the bioactivity and the MS/MS (MS^2^) data of the fractions using the B-B MN workflow [14]. Briefly, the acquired MS^2^ data was processed using MZmine2 toolbox to detect and assess relative quantification of LC-MS/MS spectral features (ions) across the chromatographic fractions. The processed data was used to calculate a bioactivity score using the Pearson correlation coefficient (*r*) between feature intensity, i.e., the molecule’s relative abundance calculated from the LC-MS peak (area under the curve) across the fraction, and the anticancer activity of each fraction. The nodes with higher *r* (>0.75) and lower *p* (<0.05) values indicated the presence of most active metabolites in fractions 5–7. Since this approach only allowed using single bioactivity data per analysis, activity results against melanoma cancer cell line A-375 was used to calculate bioactivity scores. Finally, the processed data was analyzed on the GNPS platform and visualized by Cytoscape^®^.

**Figure 2:**
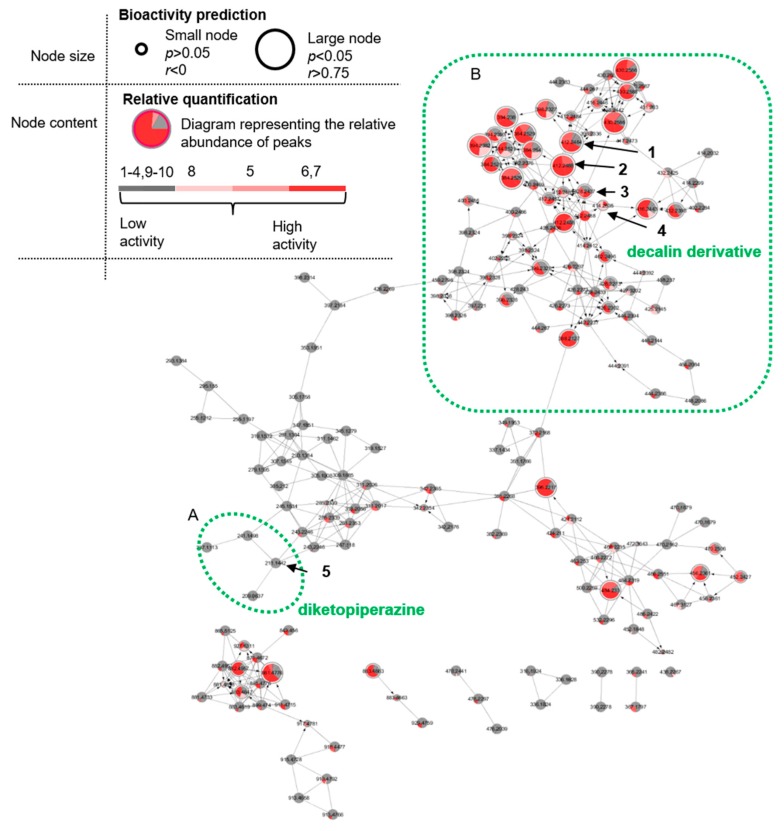
Bioactivity-based molecular networking of SPE fractions obtained from chloroform subextract of *Pyrenochaetopsis* sp. FVE-001. (**A**) Subcluster of diketopiperazine chemical family detected in MN. (**B**) Subcluster of decalin family detected in MN. **1**–**4**: Decalinoyltetramic acid derivatives, **5**: known diketopiperazine cyclo-(leu-pro).

The B-B MN of the SPE fractions obtained from the KC subextract of *Pyrenochaetopsis* sp. FVE-001 is displayed in Figure 2. A total of 175 nodes forming seven different chemical clusters were identified after removal of nodes from chromatography solvents and the medium. The node size in Figure 2 indicates the statistical significance of bioactivity score, i.e., the largest nodes represent molecules with strong Pearson correlation (*r* > 0.75) and a significance (*p* < 0.05). Since the *r* value has linear correlation between the molecule’s relative abundance and the level of the bioactivity, the B-B MN directly visualizes the relative bioactivity of the detected molecules. The relative abundance of the detected molecules in the each fraction is shown in each node with a pie chart diagram (colors corresponding to the bioactivity level of each fraction, i.e., red for samples with inhibition rate >90%, pink for samples with inhibition rate 90–75%, light pink for samples with inhibition rate 75–20% and grey for inactive samples). In total, 10.3% (18 of total 175 nodes) of molecules showed a statistically significant bioactivity score (*r* > 0.75 and *p* < 0.05). This approach enabled us to narrow down the potentially active metabolites to 18 nodes (out of 56 nodes) that were detected in the highly active fractions 5–7.

**Figure 3:**
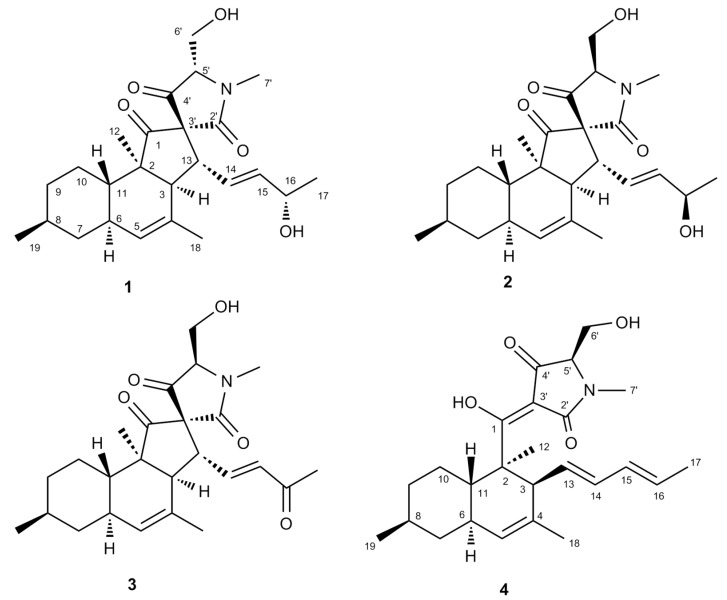
Chemical structures of compounds **1**–**4**.

A detailed analysis of the MN permitted the annotation of several fungal metabolites to different molecular families (Figure 2). The biggest cluster with 144 nodes consisted of several subclusters (e.g., **A** and **B**). The node with the precursor ion *m/z* 211.1442 ([M + H]^+^, C_11_H_19_N_2_O_2_) in subcluster **A** showed a close MS/MS spectral matching with GNPS spectral library and was annotated as the known diketopiperazine cyclo-(leu-pro) (**5**) [17]. Three related nodes in subcluster **A** at *m/z* 209.0637, 241.1498 and 237.1113 that clustered with **5** were also identified as structurally related diketopiperazines. The subcluster **B**, which contains the most of the potential bioactive metabolites revealed a node (*m/z* 414.2645 [M + H]^+^, C_25_H_36_NO_4_) suggestive of the known fungal metabolite phomasetin (**4**) [18] (Figure 3). This was further confirmed by diagnostic MS/MS fragments corresponding to the loss of the olefinic side chain of the decalin moiety (*m/z* 346.2017 and 328.1915) and the cleavage of the tetramic acid moiety (*m/z* 271.2062 and 243.2113). No additional spectral annotation could be retrieved from GNPS library for other related nodes in the subcluster **B**. However, detailed analysis of MS/MS fragmentation patterns suggested that molecules in subcluster **B** are closely related to phomasetin (**4**). The subcluster **B** consisted of the highest number of potentially active nodes (15 of total 18 nodes). Based on the significant bioactivity score of nodes at *m/z* 412.2484 (**1**) ([M − H_2_O + H]^+^) and 412.2488 (**2**) ([M − H_2_O + H]^+^) (*r* values of 0.79, 0.93 and *p* values of 2.2 × 10^-3^ and 1.30 × 10^-5^, respectively) and their close similarity to phomasetin (**4**), we carried out a targeted isolation of these putatively bioactive molecules. In addition, a related molecule with *m/z* 428.2434 [M + H]^+^ (**3**) with low bioactivity score (*r* value 0.45) was also targeted due to the high probability to be an analogue of the most active metabolites.

### 2.3. Purification and Structure Elucidation

The comprehensive analysis of the B-B MN revealed fractions 5 and 6 to have the highest relative abundance of targeted molecules. Based on this, fractions 5 and 6 were combined (106 mg altogether) and subjected to RP-HPLC separation to afford three new compounds, **1**–**3** (Figure 3). The known metabolite phomasetin (**4**) (Figure 3), which was previously identified by MS/MS based dereplication (see above), was isolated from fraction 7. The chemical structure of phomasetin (**4**) was confirmed by comparing its HRMS, NMR (Table 1 and Table 2) and [α]_D_ data with those reported in the literature [19].

**Figure 4:**
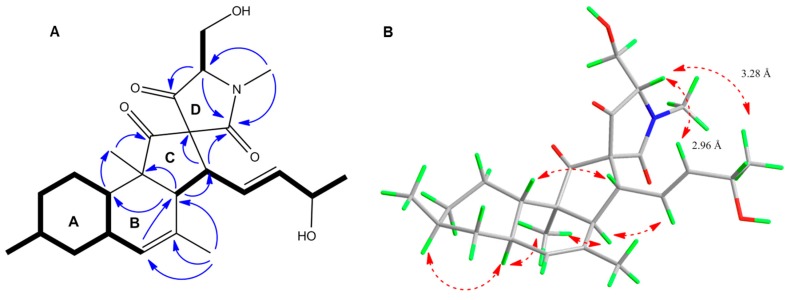
(**A**) Key COSY (bold), HMBC (blue) correlations within **1**. (**B**) NOESY (red) correlations and distances between H-5′, H-15 and H_3_-17 shown on the Chem3D optimized model of **1**.

Compound **1** was isolated as a colorless oil. HR-ESIMS spectrum of **1** contained a molecular ion peak at *m/z* 430.2592 [M + H]^+^ consistent with the molecular formula C_25_H_35_NO_5_ requiring 9 degrees of unsaturation (Figure S7). The FT-IR spectrum implied the presence of hydroxyl and carbonyl groups (3249–3554, 1683 and 1721 cm^−1^, respectively) (Figure S8). The ^13^C-NMR data (Table 2, Figure S2) comprised 25 carbon signals, including four olefinic carbons at *δ*c 127.3, 128.1, 131.8 and 138.8 along with three carbonyl groups at *δ*_C_ 168.6, 207.2 and 213.3. The ^1^H NMR and DEPT-HSQC spectra of **1** (Table 1, Figures S1 and S3) contained signals corresponding to five methyl groups, of which two are secondary (*δ*_H_ 1.19, d, *J* = 6.4 Hz, H_3_-17; *δ*_H_ 0.90, d, *J =* 6.5 Hz, H_3_-19), one tertiary (*δ*_H_ 0.98, s, H_3_-12), one olefinic (*δ*_H_ 1.73, br s, H_3_-18) and one being an *N*-methyl (*δ*_H_ 3.10, s, H_3_-7′). The DEPT-HSQC spectrum also confirmed the presence of four diastereotopic methylene protons corresponding to H_2_-7 (δ_H_ 0.86 and 1.78), H_2_-9 (δ_H_ 1.01 and 1.72), H_2_-10 (δ_H_ 1.04 and 1.42) and the oxymethylene H_2_-6′ (*δ*_H_ 4.09, dd, *J* = 12.2, 2.6 Hz and *δ*_H_ 3.92, dd, *J* = 12.3, 4.8 Hz). Further detected were ten complex methine protons, six of which appearing at *δ*_H_ 2.57 (H-3, d, *J* = 11.4 Hz), *δ*_H_ 1.43 (H-8, m), *δ*_H_ 1.82 (H-6, m), *δ*_H_ 1.63 (H-11, m), *δ*_H_ 3.44 (H-13, dd, *J* = 11.4, 8.6 Hz) and *δ*_H_ 3.57 (H-5′, dd, *J* = 4.8, 2.6 Hz). The seventh (oxy) methine proton appeared at *δ*_H_ 4.24 (H-16, m). The remaining three methine protons were olefinic; two appeared as part of an AB system at *δ*_H_ 5.75 (H-14, dd, *J* = 15.4, 8.6 Hz) and *δ*_H_ 5.71 (H-15, dd *J* = 15.4, 4.8 Hz), while the final olefinic methine (H-5) emerged as a broad singlet at *δ*_H_ 5.24 (Figure S1). The ^1^H-^1^H COSY spectrum of **1** (Figure S4) led to the establishment of three spin systems. As shown in Figure 4A, the shortest spin system belonged to a hydroxyethyl group including H-5′ and H-6′, while the second spin system was represented by a 16-hydroxypentenyl group (H-13 to H_3_-17). The largest and third COSY spin network corresponded to a substituted methylcyclohexane moiety, starting with the secondary methyl H_3_-19 and ending with the olefinic H-5 (Figure 4A and Figure S4). Diagnostic HMBC correlations (Figure 4A and Figure S5) observed from H-3 to C-2, C-4, C-11; from H-5 to C-3, C-7 and C-11; from H-7 to C-5, C-8 and C-9 and from H-11 to C-2, C-6, C-7, C-9 and C-10 permitted the establishment of the bicyclic partial structure, i.e., the unsaturated decalin moiety (rings **A**-**B**, Figure 4A). The olefinic methyl H_3_-18 was assigned to C-4 based on the substantial HMBC cross peaks seen from H_3_-18 to C-4, C-5 and C-3. The secondary methyl H_3_-19 was already placed at C-8 by COSY data and HMBC cross-peaks from H_3_-19 to C-7, C-8 and C-9 further confirmed its assignment. The tertiary methyl H_3_-12 had to be attached at C-2 on the basis of multiple HMBC correlations of H_3_-12 with C-1, C-2, C-3 and C-11. Additional correlations, i.e., COSY (between H-3 and H-13, H-13 and H-14) and HMBC (between H-3 with C-13, C-14 and C-15), confirmed the attachment of the 16-hydroxypentenyl side chain at C-3 of the decalin ring (Figure 4A, Figures S4 and S5). Further HMBC correlations from H-5′ to C-2′, C-4′ and from H_3_-7′ to C-2′and C-5′ established the terminal *N*-methyl tetramic acid structure (ring **D**) (Figure 4A and Figure S5). The long-range (HMBC) couplings detected between H-13/C-3′ and H-13/C-2′ confirmed the connectivity of tetramic acid ring to decalin moiety through a spiro carbon, C-3′ (Figure 4A and Figure S5). A key long range coupling between H_3_-12/C-1 and H_3_-12/C-2 and H-3/C-12 clearly assigned the carbonyl group (*δ*c 213.3) at C-1. This completed the final ring system, **C** (Figure 4A).

The relative configuration of the stereogenic centers within **1** was established mainly by NOESY data (Figure 4B and Figure S6). The NOESY correlations observed between H-6/H-8, H-6/H_3_-12, H_3_-12/H-3 and H-11/H-13 revealed the *trans* junction of the decalin ring, the α-orientation of H-3, H-6, H-8, H_3_-12 and the β-orientation of H-11, H-13 and H_3_-19 methyl group (Figure 4B). The large coupling constant (*J*_14,15_ = 15.4 Hz) and the NOESY correlation detected between H-13 and H-15 indicated the *E*-geometry of the double bond at Δ^14(15)^. The NOE correlations and the distances (Å) between relevant protons on the Chem3D optimized model of **1** assisted the assignment of the relative stereochemistry at C-5′ in the tetramic acid portion (Figure 4B, Table S3). The NOE cross-peaks detected between H-5′/H-15 (distance 2.96 Å) and H-5′/H-17 (distance 3.28 Å) indicated H-5′ to be β-oriented. The absolute configuration of C-16 was determined by Mosher’s ester method [20]. The compound **1** was converted to 16-(*S*)-MTPA methyl ester (**6**) and 16-(*R*)-MTPA methyl ester (**7**) using (*S*)- and (*R*)-MTPA chloride (Figures S25 and S26). The differences of ^1^H NMR chemical shifts around C-16 of **6** and **7** were measured (Table S2, Figure S29). The results of Δδ (δ*_S_*_–_δ*_R_*) for H-14 (0.08), H-15 (0.07) and H_3_-17 (−0.08) suggested the absolute configuration at C-16 to be *S*. Thus, we propose the trivial name pyrenosetin A for compound **1**.

Compound **2** was isolated as an optically active colorless oil. Its molecular formula C_25_H_35_NO_5_ deduced by HR-ESIMS (*m/z* 452.2401 [M + Na]^+^) was found to be the same as **1** (Figure S15). A close inspection of its FT-IR, 1D and 2D NMR spectra (Figures S9–S14 and S16) revealed that **2** had the same 2D structure as **1**. As depicted in Table 1 and Table 2, 1D NMR spectra of **2** however contained slight changes in chemical shifts of H-14 (**1** *δ*_H_ 5.75 and **2** *δ*_H_ 5.97) and C-14 (**1** *δ*_c_ 127.3 and **2** *δ*_C_ 130.5); H-15 (**1** *δ*_H_ 5.71 and **2** *δ*_H_ 5.50) and C-15 (**1** *δ*_c_ 138.8 and **2** *δ*_C_ 137.8); and H-16 (**1** *δ*_H_ 4.24 and **2** *δ*_H_ 4.18) and C-16 (**1** *δ*_c_ 67.9 and **2** *δ*_C_ 69.0). These differences were indicative of an opposite (*R*) stereochemistry at C-16. The absolute configuration of C-16 was investigated by Mosher’s method. The chemical shift differences [Δδ(δ*_S_*_-_δ*_R_*)] in the ^1^H-NMR of the MTPA esters (**8** and **9**) for H-14 (−0.01), H-15 (−0.13) and H_3_-17 (+0.04) suggested a 16-*R* absolute configuration, which was opposite to **1** (Table S2, Figures S27, S28 and S30). The absence of any NOESY correlation between H-5′and H-15 or between H-5′and H-17 suggested the α-orientation of H-5′. The distances calculated on the 3D structure of compound **2** (with both α- and β-epimers of H-5′) (Table S3) supported this assignment. Based on the NOESY spectrum (Figure S14), the relative configuration of all other chiral centers within **2** was identified to be the same as in compound **1.** We suggest the trivial name pyrenosetin B for compound **2**.

HR-ESIMS analysis of compound **3** revealed a molecular ion (*m/z* 428.2434 [M + H]^+^) that corresponded to a molecular formula C_25_H_33_NO_5_ (Figure S23). This requires 10 degrees of unsaturation. The FT-IR, 1D and 2D NMR data of **3** closely resembled compounds **1** and **2** (Figures S17–S22 and S24). The two most striking differences were the absence of H-16 oxymethine resonance in the ^1^H-NMR spectrum of **2**, and the appearance of an additional carbonyl singlet at *δ*_C_ 197.6 (C-16) in its ^13^C-NMR spectrum (Table 1 and Table 2, Figures S17 and S18). This clearly suggested **3** to be an oxidized analog of **1** and **2**. The conversion of the secondary methyl group H_3_-17 into a highly deshielded singlet at *δ*_H_ 2.22 (Table 1) indicated that the hydroxyl group at C-16 was replaced by a ketone function. The remarkable shift of H-14 and H-15 protons to downfield up to +0.8 and 1.0 ppm, respectively, further supported this assumption (Table 1). Clear HMBC correlations from H-14, H-15 and H_3_-17 to C-16 provided the final proof for the presence of a carbonyl function at C-16. The planar structure of **3** was confirmed by detailed analysis of ^1^H, ^13^C, DEPT-HSQC, COSY and HMBC data (Figures S17–S22). The relative configuration of the chiral centers at C-3, C-6, C-8, C-11, C-2, C-13, C-3′ and C-5′ within **3** was deduced to be the same as in **2** by analyzing its NOESY spectrum (Figure S22) and distances between proton pairs H-5′/H-15 and H-5′/H-17 (Table S3). We suggest the trivial name pyrenosetin C for compound **3**.

### 2.4. Biological Activity of Compounds 1–4

Compounds **1**–**4** were tested against the human malignant melanoma cancer cells (A-375). As shown in Table 3, compounds **1** and **2** showed the highest anticancer activity with IC_50_ values of 2.8 and 6.3 μM, respectively. Compound **3** and the known metabolite **4** were less potent (IC_50_ 140.3 and 37.3 μM). The general toxicity of the isolated metabolites was assessed against the human keratinocyte cell line HaCaT. Interestingly the IC_50_ values of **1**, **3** and **4** against HaCaT cells were very similar to their IC_50_ values against melanoma cells, indicating them to be non-selectively toxic. However, compound **2** exerted lower toxicity towards HaCaT cells (IC_50_ 35.0 μM) with a relatively better selectivity index around 5.6 (calculated by dividing IC_50_ value against HaCaT cells / IC_50_ value against A-375 cells) compared to other three fungal metabolites.

## 3. Discussion

Phylogenetic tree analysis confirmed the fungus FVE-001 to be a *Pyrenochaetopsis* sp. The genus *Pyrenochaetopsis* has previously been reported as a close relative of *Pyrenochaeta*, which is a member of the family Cucurbitariaceae. The genus *Pyrenochaetopsis* shows genetic similarity to the genus *Phoma*, but their conidiogenesis is considered to be significantly different [21,22]. The chemical machinery of the genus *Pyrenochaetopsis* has not received much attention so far. To our knowledge, only one chemical study has been performed on terrestrial soil fungus *Pyrenochaetopsis* sp., reporting the isolation of phomasetin (**4**) and two new decalinoyl tetramic acids, wakodecalines A and B [18]. A very recent study by Kato et al. (2018) has identified the biosynthetic gene cluster of phomasetin in a *Pyrenochaetopsis* sp. RK10-F058 [23]. The wakodecalines A and B showed moderate antimalarial activity against the *Plasmodium falciparum* 3D7, but were inactive against cancer cell lines [18]. Phomasetin (**4**) has been reported from few fungal genera, e.g., *Phoma* and *Pyrenochaetopsis*. It exerts anti-HIV and antitumor activities against cancer cell lines such as HeLa and HL-60 [18,19]. In this study phomasetin showed weak antitumor activity, which was associated with general toxicity.

The major drawbacks of the traditional bioassay-guided isolation process include its time consuming nature and the re-isolation of known compounds. Molecular network-based metabolomics is emerging as an efficient method in identification of chemical inventory of biological organisms and dereplication of natural products from crude extracts [11,24]. In our previous work, we used OSMAC approach to investigate chemical diversity and anticancer activity of fungi associated with *Fucus vesiculosus* [12] where we mapped the anticancer activity data of large number of fungal extracts over the MN and searched for a link between spectral molecular networks and their anticancer activity [12]. One fungus from this study that was identified herein as *Pyrenochaetopsi*s sp. FVE-001 (order Pleosporales) was selected for work-up, based on its anticancer activity. The combination of both bioassay-guided fractionation and MN approaches in the form of B-B MN allowed the rapid isolation of three new compounds **1**–**3**. As expected, pyrenosetins A (**1**) and B (**2**) had the highest anticancer activity, supporting the bioactivity scores obtained by B-B MN at fractionation stage. Pyrenosetin B (**2**) that differs from **1** by the stereochemistry of the C-16 hydroxyl group displayed less pronounced anticancer activity but also lower toxicity. The replacement of the OH group at C-16 with a keto function in pyrenosetin C (**3**) was not favorable for bioactivity, leading to a low IC_50_ value against A-375 cells. This indicates that the presence of an OH group at C-16 impacts the anticancer and toxic potential of pyrenosetins A-C.

Compounds **1**–**3** possess unique decalinoylspirotetramic acid structures characterized by a *trans*-decalin ring, a fused spiro system with a carbonyl unit (cyclopentanone) and a terminal tetramic acid moiety. Bioactive natural products containing *trans*-decalin ring are common in fungi (e.g., genera *Fusarium, Penicillium* and *Alternaria*) [25], however, molecules containing a decalin ring with a terminal tetramic acid structure linked through a spiro ring are rare in Nature. Some known examples include fusarisetins A and B from a soil-derived *Fusarium* sp., fusarisetins C-D from a marine-derived *Fusarium* sp. [26,27], altercrasins A-E from a sea urchin-derived *Alternaria* sp. [28]. Wakodecalines A and B obtained from the terrestrial soil fungus *Pyrenochaetopsis* sp. RK10-F058 possess a *N*-methylated serine moiety instead of the terminal tetramic acid ring [18]. Interestingly some *Fusarium sp.* [29] contain equisetin, the enantiomeric homolog of phomasetin, which possesses totally opposite stereochemistry in all chiral centers. Such compounds have been proposed to be mixed biosynthesis products of PKS and NRPS pathways [23]. Despite the structural similarities, biosynthesis of decalin derivatives is highly controlled by stereospecific enzymes [19,30]. A recent study by Kato et al. (2018) reported that the stereospecific enzyme *fas2* determines the formation of the *trans*-decalin ring in equisetin (and fusarisetin), while *phm7* enzyme controls the biosynthesis of the *trans*-decalin ring in the enantiomeric homolog phomasetin (**4**) [23]. In this study, we isolated phomasetin (**4**) and three new related metabolites **1**–**3** from *Pyrenochaetopsis* sp. FVE-001. This is the first chemical study carried out on a marine-derived *Pyrenochaetopsis* sp. and the second investigation performed on a member of the genus *Pyrenochaetopsis*. Although the anticancer activity the compounds **1** and **2** is associated with cytotoxicity, a medicinal chemistry approach in future may improve their potency and selectivity.

## 4. Material and Methods

### 4.1. General Procedures

NMR spectra were recorded on a Bruker AV 600 spectrometer (600 and 150 MHz for ^1^H- and ^13^C-NMR, respectively, Bruker^®^, Billerica, MA, USA) or a Bruker Avance III spectrometer (500 and 125 MHz for ^1^H- and ^13^C-NMR, respectively). The residual solvent signals were used as internal references: *δ*_H_ 7.26/*δ*_C_ 77.2 ppm (CDCl_3_), *δ*_H_ 1.94/*δ*_C_ 118.3 and *δ*_C_ 1.3 (CD_3_CN). 4-Dimethyl- 4-silapentane-1-sulfonic acid (DSS) was used as internal standard. High-resolution MS^2^ data were recorded on a Xevo G2-XS QTof Mass Spectrometer (Waters^®^, Milford, MA, USA) coupled to a Waters Acquity I-Class UPLC system). HR-ESIMS was recorded on a Bruker microTOF II-High-performance TOF-MS system equipped with an electrospray ionization source. Solid phase extraction (SPE) was performed on the Chromabond C18 SPE cartridges (6 mL/2000 mg, Macherey-Nagel, Duren, Germany). HPLC separations were performed on a VWR Hitachi Chromaster system (VWR International, Allison Park, PA, USA) consisting of a 5430 diode array detector (VWR International, Allison Park, PA, USA), a 5310 column oven, a 5260 autosampler and a 5110 pump combined in parallel with a VWR Evaporative Light Scattering Detector (ELSD 90). Routine HPLC separations were performed on semipreparative (Onyx, 100 × 10 mm, Phenomenex, Torrance, CA, USA) C18 monolithic column, analytic (SeQuant^®^, 250 × 4.6 mm, Merck, Darmstadt, Germany) ZIC-HILIC column and analytic (Synergi^TM^, 4 µm, 250 × 4.6 mm, Phenomenex) polar-RP 80 Å LC column. The water used was MilliQ-water produced by in-house Arium^®^ Water Purification Systems (Sartorius, Germany). EtOAc, *n*-hexane, methanol and acetonitrile were purchased from VWR International GmbH (Hannover, Germany). Potato extract and dextrose that were used for culture medium were purchased from Sigma-Aldrich (Schnelldorf, Germany) and from Merck, respectively. Agar was purchased from Applichem (Darmstadt, Germany). (*S*)- and (*R*)-MTPA chloride were purchased from Merck. The 3D structures of compounds **1**–**3** were obtained by using ChemBio3D Ultra 12.0 software (PerkinElmer, Waltham, MA, USA).

### 4.2. Strain Identification and Cultivation

The fungal strain was isolated from *Fucus vesiculosus* specimens collected in Falckenstein Beach (54°23′22.6” N, 10°11′26.4” E), Kiel Fjord, Baltic Sea, Germany in December 2015 [12]. The fungus was identified using ITS-5.8s rRNA sequencing to species level through building phylogenetic tree (Figure 1). The evolutionary history was inferred by using the Maximum Likelihood method based on the General Time Reversible model [31]. The percentage of trees in which the associated taxa clustered together is shown next to the branches. Initial tree(s) for the heuristic search were obtained automatically by applying Neighbor-Join and BioNJ algorithms to a matrix of pairwise distances estimated using the Maximum Composite Likelihood (MCL) approach, and then selecting the topology with superior log likelihood value. A discrete Gamma distribution was used to model evolutionary rate differences among sites (5 categories (+G, parameter = 0.8198)). The rate variation model allowed for some sites to be evolutionarily invariable ([+I], 37.97% sites). The tree is drawn to scale, with branch lengths measured in the number of substitutions per site. The analysis involved 14 nucleotide sequences. Evolutionary analyses were conducted in MEGA7 [32]. The fungal sample was deposited at the GEOMAR-Biotech laboratory (Kiel, Germany). The strain was cultured on potato dextrose agar (PDA: potato extract 4 g, dextrose 20 g, agar 15g for 1 L, pH 5.6). After 3 days of pre-culturing, the conidia was inoculated in 500 mL cylindrical flasks containing 100 mL of seed medium (PDM: potato extract 4 g, dextrose 20 g for 1 L; pH 5.6) incubated at 22 °C for 7 days on a rotary shaker at 120 rpm.

### 4.3. Extraction and Isolation

The 12 L of culture broth was partitioned with the same volume of EtOAc twice. The resulting organic extract was evaporated *in vacuo* to afford light yellow oily extract (2.66 g). The crude extract was subjected to a modified Kupchan partition to yield three subextracts, namely *n*-hexane (KH, 1.99 g), CHCl_3_ (KC, 345.4 mg) and aqueous MeOH (KM, 68.7 mg). Briefly, the crude extract was dissolved in 500 mL aqueous methanol (90% MeOH) and partitioned against *n*-hexane (KH, 2 × 500 mL). The MeOH content of the aq. MeOH phase was increased to 30% before partitioning against CHCl_3_ (KC, 2 × 500 mL). The KC subextract showed relatively high anticancer bioactivity against all five cancer cell lines (>75% inhibition rate at 100 µg/mL) and was fractionated on a Chromabond C18 SPE cartridge. A 10% stepwise gradient elution with MeOH: H_2_O mixtures (0% to 100%) afforded 11 fractions (fraction 0–10). Anticancer activity was tracked to fractions 5–7. The fraction 5 (92.7 mg) and fraction 6 (13.3 mg) were combined and subjected to semi-preparative RP–HPLC, eluting with MeCN: H_2_O (40% isocratic MeCN over 28 min, and gradual increase to 60% by 40 min, flow 3.0 mL/min) to yield 7 subfractions (fraction 5.1 to 5.7). Subfraction 5.4 contained the pure compound **1** (2.4 mg, t_R_ 16.5 min). Fraction 5.7 (3.6 mg) was rechromatographed by RP-HPLC (isocratic mixture H_2_O: MeCN (45:55, flow 1.0 mL/min) to yield **2** (1.2 mg, t_R_ 8.0 min) and **3** (0.8 mg, t_R_ 9.5 min). The fraction 7 (25.4 mg) was subjected to a semi-prep. RP–HPLC (65% isocratic MeCN over 30 min, and gradual decrease to 60% MeCN to 60%MeCN by 35 min, flow 3.0 mL/min) to yield 5 subfractions (fraction 7.1–7.5). Fraction 7.4 (12.9 mg) was separated on HPLC equipped with an analytical HPLC column (Synergi^TM^, 250 × 4.6 mm, Phenomenex) using MeCN:H_2_O (50% isocratic MeCN over 6 min and gradual increase to 78% MeCN by 12 min (flow 1.0 mL/min)) to yield **4** (1.4 mg, t_R_ 6.5 min) in a pure state.

*Pyrenosetin A* (**1**): Colorless oil; [α]${}_{D}^{22}$ +30 (*c* 0.10, MeOH); IR (oil) *v*_max_ 3554, 3249, 2952, 2875, 1721, 1683, 1456, 1378, 1271 cm^−1^; ^1^H-NMR (CDCl_3_, 600 MHz) and ^13^C-NMR (CDCl_3_, 150 MHz), Table 1 and Table 2; HR-ESIMS found *m/z* [M + H]^+^ 430.2592, C_25_H_36_NO_5_, calculated for 430.2588.

*Pyrenosetin B* (**2**): Colorless oil; [α]${}_{D}^{22}$ +13 (*c* 0.10, MeOH); IR (oil) *v*_max_ 3550, 3185, 2944, 2862, 1726, 1675, 1456, 1378, 1249 cm^−1^; ^1^H-NMR (CDCl_3_, 600 MHz) and ^13^C-NMR (CDCl_3_, 150 MHz), Table 1 and Table 2; HR-ESIMS found *m/z* [M + Na]^+^ 452.2401 C_25_H_35_NO_5_Na, calculated for 452.2407.

*Pyrenosetin C* (**3**): Colorless oil; [α]${}_{D}^{22}$ +12 (*c* 0.10, MeOH); IR (oil) *v*_max_ 3559, 3259, 2952, 2858, 1726, 1683, 1451, 1369, 1253 cm^−1^; ^1^H-NMR (CDCl_3_, 600 MHz) and ^13^C-NMR (CDCl_3_, 150 MHz), Table 1 and Table 2; HR-ESIMS found *m/z* [M + H]^+^ 428.2434, C_25_H_34_NO_5_, calculated for 428.2431.

*Phomasetin* (**4**): Colorless oil; [α]${}_{D}^{22}$ +116 (*c* 0.15, CDCl_3_); ^1^H-NMR (CD_3_CN, 600 MHz) and ^13^C- NMR (CD_3_CN, 150 MHz), Table 1 and Table 2; HR-ESIMS found *m/z* [M + H]^+^ 414.2645, C_25_H_36_NO_4_, calculated for 414.2639.

### 4.4. UHPLC-QToF-MS/MS Analysis and Molecular Networking

The SPE fractions derived from the KC subextract were analyzed on an ACQUITY UPLC I-Class System coupled to the Waters^®^ Xevo G2-XS QToF Mass Spectrometer equipped with an electrospray ionization (ESI) source operating with a mass range of *m/z* 50–1600 Da. The 0.1 mg/mL MeOH solution of each fraction was filtered through a 0.2 µm PTFE syringe filter (Carl Roth, Karlsruhe, Germany) and then injected (injection volume: 0.2 µL) into the system that equipped with Acquity UPLC HSS T3 column (High Strength Silica C18, 1.8 µm, 100 × 2.1 mm I.D., Waters^®^) operating at 40 °C. Separation was achieved with a binary LC solvent system controlled by MassLynx^®^ (version 4.1) using mobile phase A (99%) water/ 0.1 % formic acid (ULC/MS grade) and B 99.9% ACN/ 0.1% formic acid (ULC/MS grade), pumped at a rate of 0.6 mL/ min with the following gradient: initial, 1% B; 0.0–6.0 min to 30% B; 6.0–11.5 min to 100% B; 11.5–13.5 min 100% B, and a column reconditioning phase until 15 min.

ESI conditions were set with the capillary voltage at 0.8 kV, sample cone voltage at 40.0 V, source temperature at 150 °C, desolvation temperature at 550 °C, cone gas flow in 50 L/h and desolvation gas flow in 1200 L/h. MS/MS setting was a ramp collision energy (CE): low CE from 6 eV to 60 eV and high CE from 9 eV to 80 eV. As a control, solvent (methanol) was injected. MassLynx^®^ (Waters^®^, V4.1) was used to analyze the achieved MS and MS^2^ data.

The raw data were converted to mzXML file format using MSConvert (version 3.0.10051, Vanderbilt University, Nashville, TN, USA). The resulting mzXML data were processed in MZmine2 (version 2.32). Mass lists were created using the mass detection module with a noise level of 1500 counts for MS^1^ and 10 counts for MS^2^. The chromatogram builder module was used to create peak lists with a scan retention time from 0.01 min to 13.0 min, a minimum retention time of 0.01 min, a minimum peak height of 10,000 counts and an *m/z* tolerance of 0.01 Da or 10 ppm. The peak lists were deconvoluted using the local minimum search algorithm with the following parameters: chromatographic threshold 0.01%, search minimum in RT range 0.20, minimum relative height 0.01%, minimum absolute height 1000 counts, minimum ratio of peak top/edge 2, peak duration range 0.01–3 min. For MS^2^ scan pairing, the *m/z* range was set to 0.05 Da and retention time range to 0.2 min. Furthermore, the deconvoluted peak lists were deisotoped with the following parameters: *m/z* tolerance 0.02 or 10 ppm, retention time tolerance 0.01 min and maximum charge 2. The deisotoped peak lists were aligned through using the Join aligner module with an *m/z* tolerance of 0.01 Da or 10 ppm and a retention time tolerance of 0.2 min. Weight for *m/z* and retention time were both set to 50. The aligned peak list was filtered to exclude peaks derived from solvent and peaks with *m/z* lower than 125 Da. The peak ID’s were lastly reset and the peak list was exported as mgf file for GNPS analysis. The reset peak list was exported as CSV table file for bioactivity score analysis.

The adjusted mgf files were uploaded to GNPS for molecular networking analysis. A molecular network was created with the feature based molecular networking work flow (https://ccms-ucsd.github.io/GNPSDocumentation/featurebasedmolecularnetworking/) on the GNPS website (http://gnps.ucsd.edu) [33]. The data was filtered by removing all MS/MS fragment ions within ±17 Da of the precursor *m/z*. MS/MS spectra were window filtered by choosing only the top 6 fragment ions in the ±50 Da window throughout the spectrum. The precursor ion mass tolerance was set to 0.02 Da and a MS/MS fragment ion tolerance of 0.02 Da. A network was then created where edges were filtered to have a cosine score above 0.65 and more than 6 matched peaks. Further, edges between two nodes were kept in the network if and only if each of the nodes appeared in each other’s respective top 10 most similar nodes. Finally, the maximum size of a molecular family was set to 250, and the lowest scoring edges were removed from molecular families until the molecular family size was below this threshold. The spectra in the network were then searched against GNPS spectral libraries. The library spectra were filtered in the same manner as the input data. All matches kept between network spectra and library spectra were required to have a score above 0.65 and at least 6 matched peaks. The CSV table generated from MZmine2 (ver. 2.32) was input to R studio (version 1.1.463) to calculate the bioactivity score based on the workflow reported by Nothias et al. [14]. The obtained bioactivity score list and GNPS data were downloaded and imported into Cytoscape^®^ (version 3.5.1, Institute for Systems Biology, Seattle, WA, USA) to generate the bioactivity-based molecular networking.

### 4.5. Mosher’s Esterification

#### 4.5.1. Preparation of 16-(S) MTPA Ester 6 and 16-(R)-MTPA Ester 7

To a solution of **1** (1.1 mg, 2.1 μmol) in abs. pyridine (0.3 mL), (*S*)-MTPA chloride (3.2 mg, 12.6 μmol) was added, and the reaction mixture was stirred at room temperature for 12 h. Subsequently 1.0 mL water was added to the reaction mixture and extracted using CH_2_Cl_2_. The organic layer was evaporated under reduced pressure, and the residue was purified by RP-HPLC eluted with MeCN:H_2_O (gradient from 50:50 to 100:0 in 10 min, 100:0 in 10 to 15 min, and from 100:0 to 50:50 in 15 to 21 min, flow 1.0 mL/min) to afford 16-(*S*)-MTPA ester **6** (0.7 mg, t_R_ 12.0 min) as a colorless oil. The 16-(*R*)-MTPA ester **7** was prepared from **1** using (*R*)-MTPA chloride in pyridine employing the same procedure. The 16-(*R*)-MTPA ester **7** (0.5 mg, t_R_ 11.9 min) was a colorless oil. Both **6** and **7** were dissolved in CDCl_3_ and analysed by ^1^H-NMR spectroscopy.

#### 4.5.2. Preparation of 16-(S) MTPA Ester 8 and 16-(R)-MTPA Ester 9

Compound **8** and **9**, respectively, were individually prepared from **2** using either with (*S*)-MTPA chloride) or (*R*)-MTPA chloride. The same procedure used for preparation of **6** was employed. Both 16-(*S*)-MTPA ester **8** (0.3 mg, t_R_ 11.3 min) and 16-(*R*)-MTPA ester **9** (0.3 mg, t_R_ 11.2 min) were colorless oils. They were dissolved in CDCl_3_ and analysed by ^1^H-NMR spectroscopy.

### 4.6. Bioactivity Assays

The crude extracts were initially tested in vitro against 5 human cancer cell lines: colorectal adenocarcinoma cell line HT-29 (DSMZ, Braunschweig, Germany), malignant melanoma cell line A-375 (CLS, Eppelheim, Germany), colon cancer cell line HCT-116 (DSMZ), lung carcinoma cell line A-549 (CLS, Eppelheim, Germany), human breast cancer line MB-231 (CLS), and the non-cancerous human keratinocyte line HaCaT (CLS) at a concentration of 100 µg/mL. The antitumoral activity of the crude extracts was evaluated by monitoring the metabolic activity using the CellTiterBlue Cell Viability Assay (Promega, Mannheim, Germany). HT-29 and HaCaT cells were cultivated in RPMI medium, A-549 and MB-231 cells in DMEM: Ham’s F12 medium (1:1) supplemented with 15 mM HEPES and A-375 and HCT-116 cells in DMEM medium supplemented with 4.5 g/L D-Glucose and 110 mg/L sodium pyruvate. All media were supplemented with L-Glutamine, 10% fetal bovine serum, 100 U/mL penicillin, and 100 mg/mL streptomycin. The cultures were maintained at 37 °C under a humidified atmosphere and 5% CO_2_. The cell lines were transferred every 3 or 4 days. For experimental procedure, the cells were seeded in 96 well plates at a concentration of 10,000 cells per well. A stock solution of 40 mg/mL in DMSO was prepared of each extract. After 24 h incubation, the medium was removed from the cells and 100 µL fresh medium containing the test samples was added. Each sample was prepared in duplicate once. Doxorubicin as a standard therapeutic drug was used as positive control, 0.5% DMSO and growth media were used as controls. Following compound addition, plates were cultured at 37 °C for 24 h. Afterwards, the assay was performed according to the manufacturer’s instructions and measured using the microplate reader Tecan Infinite M200 at excitation 560 nm and emission of 590 nm. For determination of IC_50_ values, a dilution series of the extracts was prepared and tested, as described before for the crude extract. The IC_50_ values were calculated by Excel as the concentration that shows 50% inhibition of the viability on the basis of a negative control (no compound) and compared with the positive control (doxorubicin).

## Figures and Tables

**Table 1:** ^1^H NMR data of compounds **1**–**4** at 600 MHz.

No.	1 ^a^	2 ^a^	3 ^a^	4 ^b^
	δ_H_, Mult (*J* in Hz)	δ_H_, Mult (*J* in Hz)	δ_H_, Mult (*J* in Hz)	δ_H_, Mult (*J* in Hz)
1	-	-	-	-
2	-	-	-	-
3	2.57, d (11.4)	2.73, d (11.4)	2.66, d (11.3)	3.17, br d (9.4)
4	-	-	-	-
5	5.24, br s	5.22, br s	5.28, br.s	5.22, br s
6	1.82, m	1.82, m	1.83, m	1.84, m
7	1.78, m	1.80, m	1.82, m	1.82, m
	0.86, m	0.84, m	0.88, m	0.86, m
8	1.43, m	1.44, m	1.44, m	1.51, m
9	1.72, m	1.73, m	1.73, m	1.76, m
	1.01, m	0.93, m	0.99, m	1.04, m
10	1.42, m	1.40, m	1.41, m	1.97, m
	1.04, m	1.07, qd (12.8,3.4)	1.04, m	1.06, m
11	1.63, m	1.42, m	1.64, td (11.0, 2.7)	1.64 m
12	0.98, s	1.00, s	1.01, s	1.38, br s
13	3.44, dd (11.4, 8.6)	3.26, dd (11.4, 9.4)	3.57, dd (11.4, 9.8)	5.27, dd (13.6, 10.5)
14	5.75, dd (15.4, 8.6)	5.97, dd (15.3, 9.4)	6.85, dd (15.9, 9.8)	5.78, dd (14.6, 10.5)
15	5.71, dd (15.4 4.8)	5.50, dd (15.3, 8.0)	6.18, d (15.9)	5.91, t (12.8)
16	4.24, m	4.18, m	-	5.56, dq (14.2, 6.8)
17	1.19, d (6.4)	1.19, d (6.2)	2.22, s	1.67, d (6.8)
18	1.73, br s	1.69, br s	1.68, br s	1.55, t (1.9)
19	0.90, d (6.5)	0.91, d (6.5)	0.90, d (6.2)	0.91, d (6.5)
2′	-	-	-	-
3′	-	-	-	-
4′	-	-	-	-
5′	3.57, dd (4.8, 2.6)	3.94, dd (2.7, 1.9)	3.61, dd (4.9, 2.7)	3.61, t (2.7)
6′	4.09, dd (12.2, 2.6)	4.08, m	4.10, m	3.87, m
	3.92, dd (12.3, 4.8)	3.86, dd (12.4, 2.7)	3.94, m	3.81, m
7′	3.10, s	3.07, s	3.11, s	2.97, brs
6′-OH			2.74, m	

^a^ Recorded in CDCl_3_, ^b^ Recorded in CD_3_CN.

**Table 2:** ^13^C NMR data of compounds **1**–**4** at 150 MHz.

Position	1 ^a^	2 ^a^	3 ^a^	4 ^b^
	δ_C_	δ_C_	δ_C_	δ_C_
1	213.3 (C)	209.8 (C)	212.1 (C)	197.6 (C)
2	54.8 (C)	54.1 (C)	54.7 (C)	49.9 (C)
3	53.6 (CH)	52.8 (CH)	53.6 (CH)	50.2 (CH)
4	131.8 (C)	132.3 (C)	130.9 (C)	132.4 (C)
5	128.1 (CH)	127.6 (CH)	128.8 (CH)	127.1 (CH)
6	37.6 (CH)	37.6 (CH)	37.6 (CH)	40.1 (CH)
7	41.9 (CH_2_)	42.0 (CH_2_)	41.8 (CH_2_)	43.1 (CH_2_)
8	33.0 (CH)	32.9 (CH)	32.9 (CH)	34.3 (CH)
9	35.3 (CH_2_)	35.3 (CH_2_)	35.2 (CH_2_)	36.6 (CH_2_)
10	25.3 (CH_2_)	25.3 (CH_2_)	25.2 (CH_2_)	29.0 (CH_2_)
11	37.4 (CH)	38.0 (CH)	37.4 (CH)	40.6 (CH)
12	15.0 (CH_3_)	15.2 (CH_3_)	15.2 (CH_3_)	14.2 (CH_3_)
13	51.5 (CH)	51.0 (CH)	50.6 (CH)	131.6 (CH)
14	127.3 (CH)	130.5 (CH)	144.4 (CH)	133.4 (CH)
15	138.8 (CH)	137.8 (CH)	133.9 (CH)	132.1 (CH)
16	67.9 (CH)	69.0 (CH)	197.6 (C)	129.2 (CH)
17	23.5 (CH_3_)	22.9 (CH_3_)	27.6 (CH_3_)	18.1 (CH_3_)
18	24.2 (CH_3_)	23.9 (CH_3_)	23.7 (CH_3_)	22.5 (CH_3_)
19	22.4 (CH_3_)	22.4 (CH_3_)	22.4 (CH_3_)	22.7 (CH_3_)
2′	168.6 (C)	168.6 (C)	167.8 (C)	178.1 (C)
3′	73.1 (C)	73.8 (C)	72.7 (C)	101.6 (C)
4′	207.2 (C)	205.0 (C)	206.4 (C)	191.5 (C)
5′	69.8 (CH)	69.4 (CH)	69.8 (CH)	68.8 (CH)
6′	60.3 (CH_2_)	58.3 (CH_2_)	60.3 (CH_2_)	59.6 (CH_2_)
7′	28.3 (CH_3_)	27.7 (CH_3_)	28.5 (CH_3_)	27.4 (CH_3_)

^a^ Recorded in CDCl_3_, ^b^ Recorded in CD_3_CN.

**Table 3:** In vitro activity of compounds **1**–**4** against human malignant melanoma cell line (A-375) and non-cancerous keratinocyte cell line (HaCaT). The IC_50_ values are in μM. Positive control: Doxorubicin.

Compound	A-375 Cells	HaCaT Cells
1	2.8 (±0.0)	4.2 (±0.0)
2	6.3 (±0.0)	35.0 (±0.0)
3	140.3 (±0.9)	142.9 (±1.4)
4	37.3 (±0.1)	45.0 (±0.2)
Positive control	0.6 (±0.0)	22.1 (±2.9)

## Supplementary Materials

The supplementary are available online at https://www.mdpi.com/1660-3397/18/1/47/s1, Bioactivity results of different KC fractions against cancer and HaCaT cell lines; NMR, HR-ESIMS and FT-IR spectra of compounds **1**–**3**. ^1^H NMR spectra of MTPA esters **6**–**9**.
