# Supplementary material for: Pyrenosetins A–C, New Decalinoylspirotetramic Acid Derivatives Isolated by Bioactivity-Based Molecular Networking from the Seaweed-Derived Fungus Pyrenochaetopsis sp. FVE-001
Source: Mar Drugs. 2020 Jan 11;18(1):47. doi: 10.3390/md18010047 (PMC7024310; doi:10.3390/md18010047)

Supporting Information

Pyrenosetins A–C, New Decalinoylspirotetramic Acid Derivatives Isolated by Bioactivity-Based Molecular Networking from the Seaweed-Derived Fungus *Pyrenochaetopsis* sp. FVE-001

Bicheng Fan [1], Pradeep Dewapriya [1], Fengjie Li [1], Martina Blümel [1] and Deniz Tasdemir [1,2,*]

[1] GEOMAR Centre for Marine Biotechnology (GEOMAR-Biotech), Research Unit Marine Natural Products Chemistry, GEOMAR Helmholtz Centre for Ocean Research Kiel, Am Kiel-Kanal 44, 24106 Kiel, Germany; bfan@geomar.de (B.F.); pdewapriya@geomar.de (P.D.); fli@geomar.de (F.L.); mbluemel@geomar.de (M.B.)

[2] Faculty of Mathematics and Natural Sciences, Kiel University, Christian-Albrechts-Platz 4, 24118 Kiel, Germany

* Correspondence: dtasdemir@geomar.de; Tel.: +49-431-600-4430

<u>**List of Tables**</u>

Table S1. In vitro anticancer activity (%) of Kupchan subextracts (KH, KC, KM) and SPE fractions against cancer cell lines (A-375, A-549, HT-29, HCT-116, MB-231) and non-cancerous HaCaT cell line.

Table S2. The $\Delta\delta(\delta_S-\delta_R)$ data for the *S*- and *R*-MTPA esters **6-9** in ^{1}H NMR ($CDCl_3$, 500 MHz).

Table S3. The distance (Å) between protons H-5′, H-15 and H_3-17 in the tetramic acid portion of the compounds **1**-**3**. The red marking indicates the assigned relative stereochemistry based on the measured distances allowing observable NOE correlations (up to 4 Å) between relevant protons.

<u>**List of Figures**</u>

Figure S1. ^{1}H NMR spectrum of compound **1** (600 MHz, $CDCl_3$).

Figure S2. ^{13}C NMR spectrum of compound **1** (150 MHz, $CDCl_3$).

Figure S3. DEPT-HSQC spectrum of compound **1** (600 MHz, $CDCl_3$).

Figure S4. COSY spectrum of compound **1** (600 MHz, $CDCl_3$).

Figure S5. HMBC spectrum of compound **1** (600 MHz, $CDCl_3$).

Figure S6. NOESY spectrum of compound **1** (600 MHz, $CDCl_3$).

Figure S7. HR-ESIMS spectrum of compound **1**.

Figure S8. FT-IR spectrum of compound **1**.

Figure S9. ^{1}H NMR spectrum of compound **2** (600 MHz, $CDCl_3$).

Figure S10. ^{13}C NMR spectrum of compound **2** (150 MHz, $CDCl_3$).

Figure S11. DEPT-HSQC spectrum of compound **2** (600 MHz, $CDCl_3$).

Figure S12. COSY spectrum of compound **2** (600 MHz, $CDCl_3$).

Figure S13. HMBC spectrum of compound **2** (600 MHz, $CDCl_3$).

Figure S14. NOESY spectrum of compound **2** (600 MHz, $CDCl_3$).

Figure S15. HR-ESIMS spectrum of compound **2**.

Figure S16. FT-IR spectrum of compound **2**.

Figure S17. ^1H NMR spectrum of compound **3** (600 MHz, $CDCl_3$).

Figure S18. ^{13}C NMR spectrum of compound **3** (150 MHz, $CDCl_3$).

Figure S19. DEPT-HSQC spectrum of compound **3** (600 MHz, $CDCl_3$).

Figure S20. COSY spectrum of compound **3** (600 MHz, $CDCl_3$).

Figure S21. HMBC spectrum of compound **3** (600 MHz, $CDCl_3$).

Figure S22. NOESY spectrum of compound **3** (600 MHz, $CDCl_3$).

Figure S23. HR-ESIMS spectrum of compound **3**.

Figure S24. FT-IR spectrum of compound **3**.

Figure S25. ^1H NMR spectrum of 16-(*S*)-MTPA ester **6** (500 MHz, $CDCl_3$).

Figure S26. ^1H NMR spectrum of 16-(*R*)-MTPA ester **7** (500 MHz, $CDCl_3$).

Figure S27. ^1H NMR spectrum of 16-(*S*)-MTPA ester **8** (500 MHz, $CDCl_3$).

Figure S28. ^1H NMR spectrum of 16-(*R*)-MTPA ester **9** (500 MHz, $CDCl_3$).

Figure S29. $\Delta\delta(\delta_S\text{-}\delta_R)$ values (ppm) obtained from 16-MTPA esters (**6** and **7**) of compound **1**.

Figure S30. $\Delta\delta(\delta_S\text{-}\delta_R)$ values (ppm) obtained from 16-MTPA esters (**8** and **9**) of compound **2.**

Table S1. In vitro anticancer activity (%) of Kupchan subextracts (KH, KC, KM) and SPE fractions against cancer cell lines (A-375, A-549, HT-29, HCT-116, MB-231) and non-cancerous HaCaT cell line.

	Cell growth inhibition (%)					
	A-375 (100 µg/ml)	A-549 (100 µg/ml)	HT-29 (100 µg/ml)	HCT-116 (100 µg/ml)	MB-231 (100 µg/ml)	HaCaT (100 µg/ml)
KH	31	0	29	0	0	31
KC	98	99	99	76	99	66
KM	0	0	0	0	0	0
KC Fr.0	0	0	0	0	0	0
KC Fr.1	0	0	0	0	0	0
KC Fr.2	0	0	0	0	0	0
KC Fr.3	0	0	0	0	0	0
KC Fr.4	0	0	0	0	0	0
KC Fr.5	85	54	99	0	63	44
KC Fr.6	99	99	99	99	99	99
KC Fr.7	99	99	99	99	99	99
KC Fr.8	43	0	0	0	0	0
KC Fr.9	0	0	0	0	0	0
KC Fr.10	0	0	0	0	0	0

Table S2. The $\Delta\delta(\delta_{S}-\delta_{R})$ data for the *S*- and *R*-MTPA esters **6–9** in ^{1}H NMR ($CDCl_3$, 500 MHz).

	**6 (*S*)**	**7 (*R*)**	$\Delta\delta_{S-R}$	**8 (*S*)**	**9 (*R*)**	$\Delta\delta_{S-R}$
**C**	δ_H, mult (*J* in Hz)	δ_H, mult (*J* in Hz)		δ_H, mult (*J* in Hz)	δ_H, mult (*J* in Hz)	
13	3.39, m	3.35, dd (11.5, 9.5)	0.04	3.27, dd (11.3, 9.7)	3.28, dd (11.4, 9.4)	-0.01
14	5.92, dd (15.5, 9.5)	5.84, dd (15.4, 9.8)	0.08	6.09, dd (14.8, 9.7)	6.10, dd (15.3, 9.7)	-0.01
15	5.65, dd (15.5, 6.4)	5.58, dd (15.4, 6.4)	0.07	5.39, dd (14.9, 7.8)	5.52, dd (15.3, 7.6)	-0.13
16	5.46, m	5.45, m	0.01	5.34, m	5.38, m	-0.04
17	1.22, d (6.5)	1.30, d (6.5)	-0.08	1.31, d (6.3)	1.27, d (6.5)	0.04

Table S3. The distance (Å) between protons H-5′, H-15 and H_3-17 in the tetramic acid portion of the compounds **1**-**3**. The red marking indicates the assigned relative stereochemistry based on measured the distances allowing observable NOE correlations (up to 4 Å) between relevant protons.

Compd	2D Structure	3D model	H-5′ orientation	Distance H-5′/H-15	NOE H-5′/H-15	Distance H-5′/H_3-17	NOE H-5′/H_3-17
1 (5′-β)	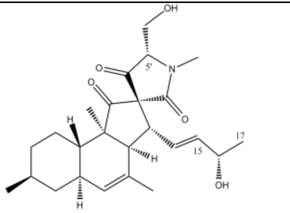	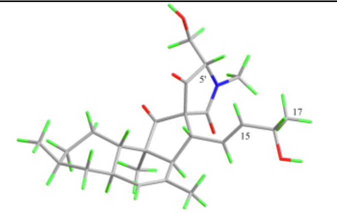	β	2.96 Å	YES	3.28 Å	YES
1 (5′-α)	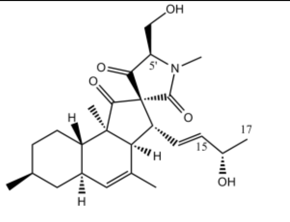	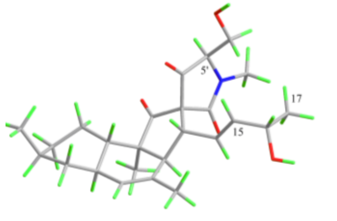	α	4.83 Å		5.07 Å	
2 (5′-β)	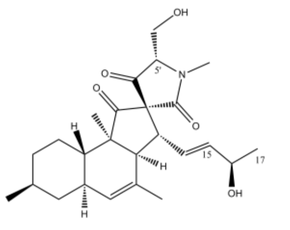	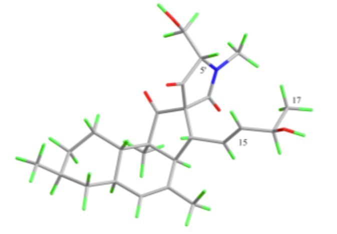	β	2.88 Å		3.33 Å	
2 (5′-α)	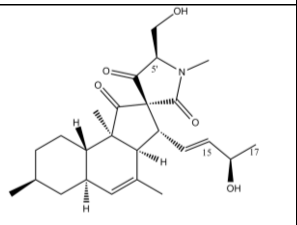	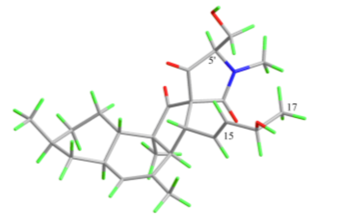	α	5.14 Å	NO	6.84 Å	NO
3 (5′-β)	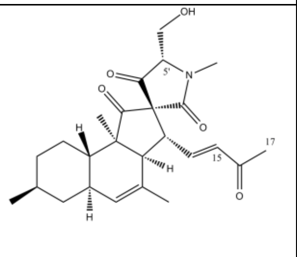	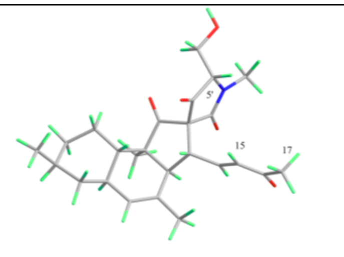	β	3.80 Å		5.11 Å	
3 (5′-α)	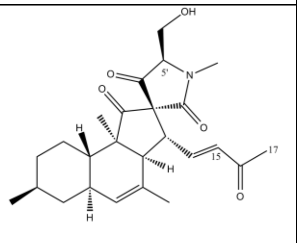	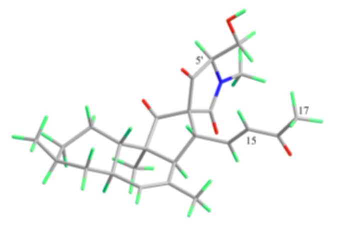	α	5.41 Å	NO	6.77 Å	NO

Figure S1. ^{1}H NMR spectrum of compound **1** (600 MHz, $CDCl_3$).

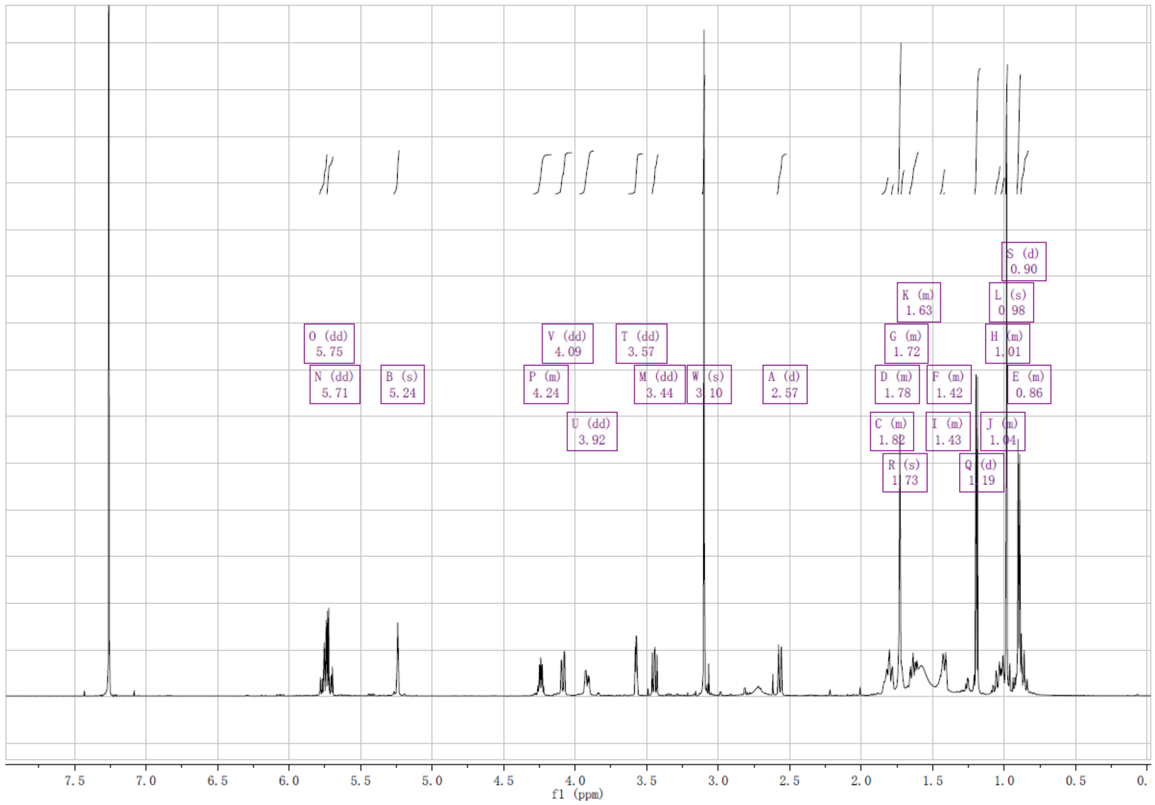

Figure S2. ^{13}C NMR spectrum of compound **1** (150 MHz, $CDCl_3$).

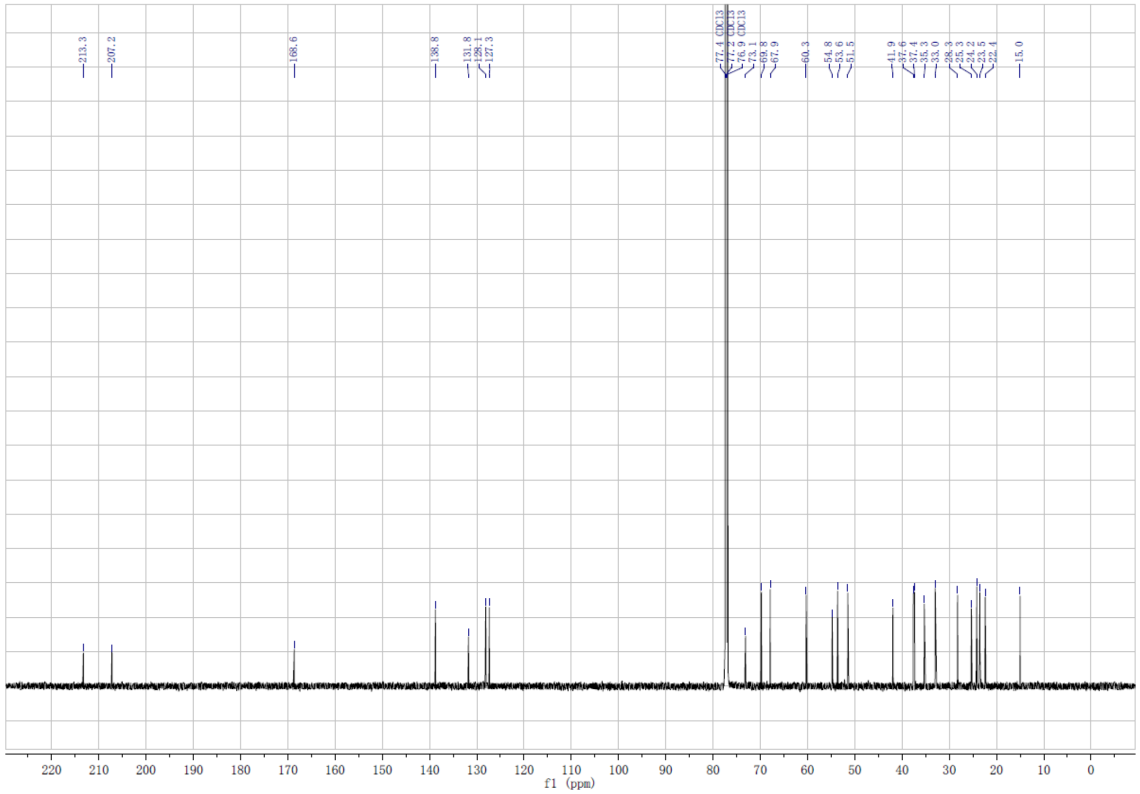

Figure S3. DEPT-HSQC spectrum of compound **1** (600 MHz, CDCl3).

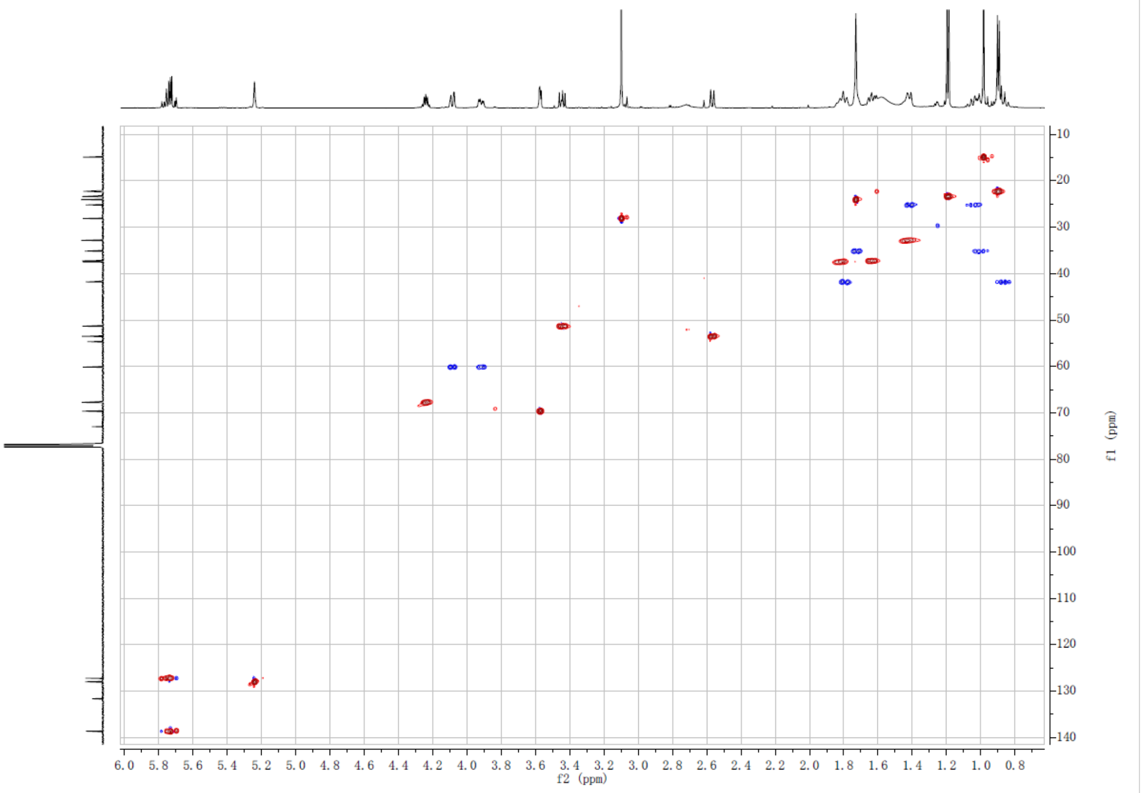

Figure S4. COSY spectrum of compound **1** (600 MHz, CDCl3).

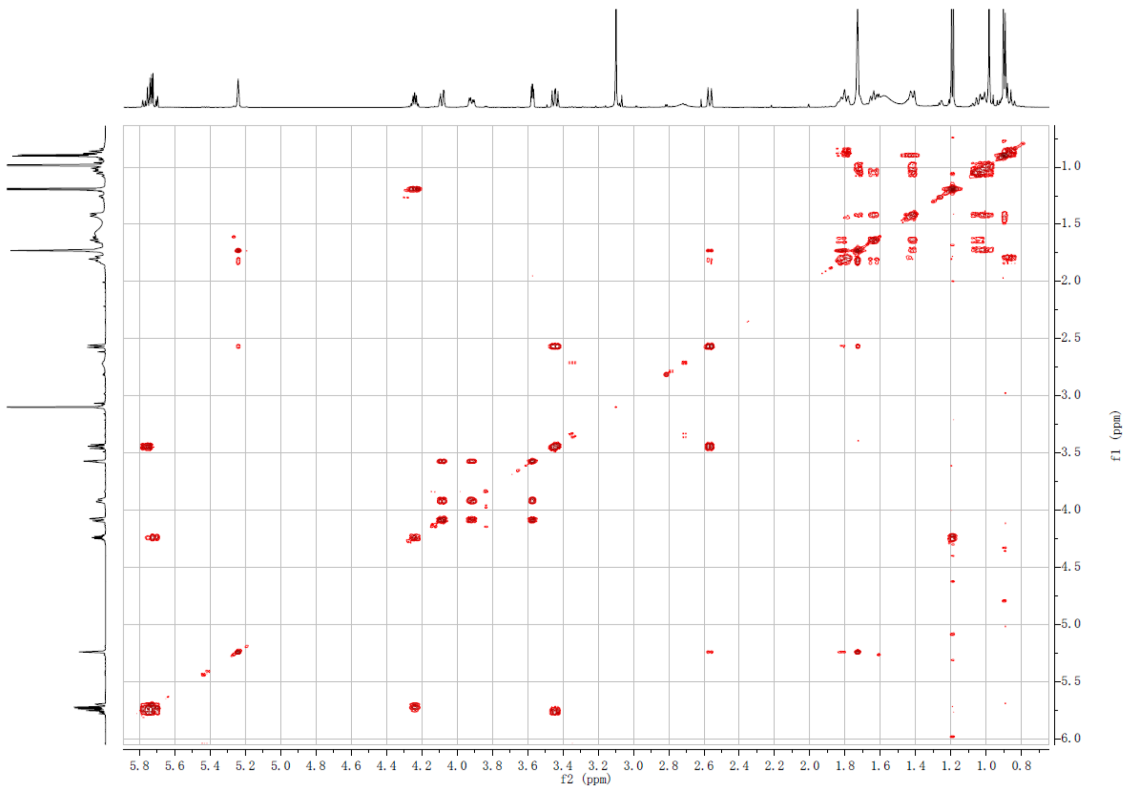

Figure S5. HMBC spectrum of compound **1** (600 MHz, $CDCl_3$).

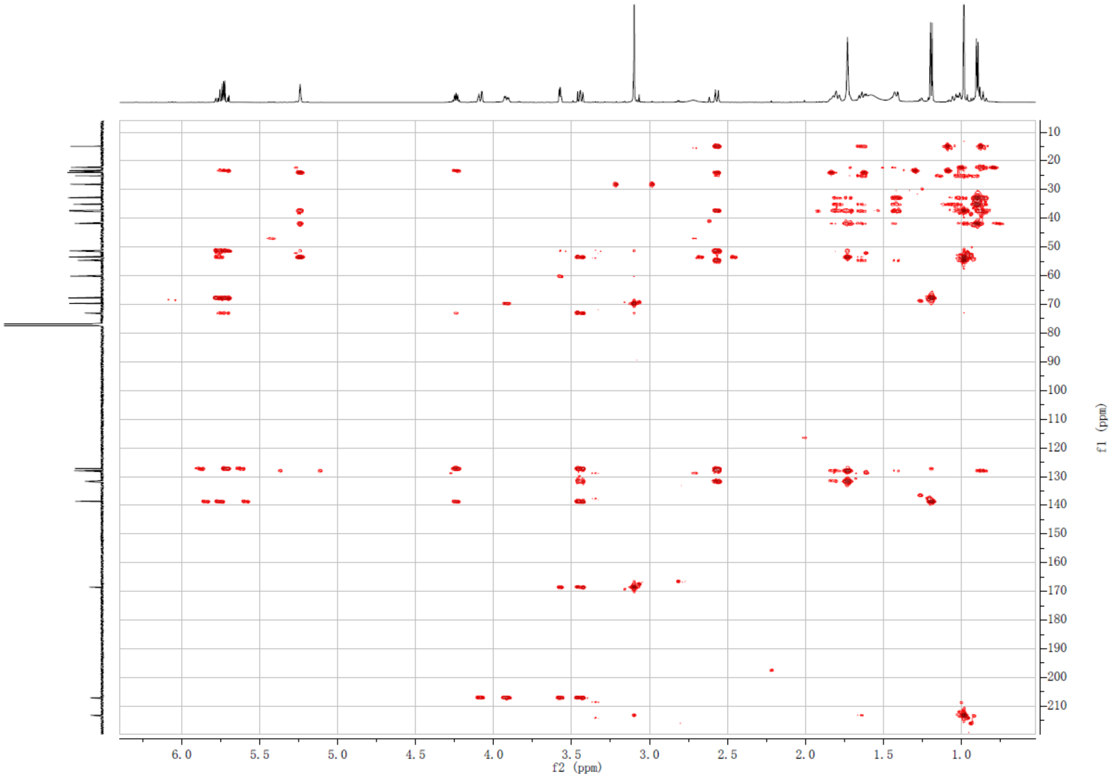

Figure S6. NOESY spectrum of compound **1** (600 MHz, $CDCl_3$).

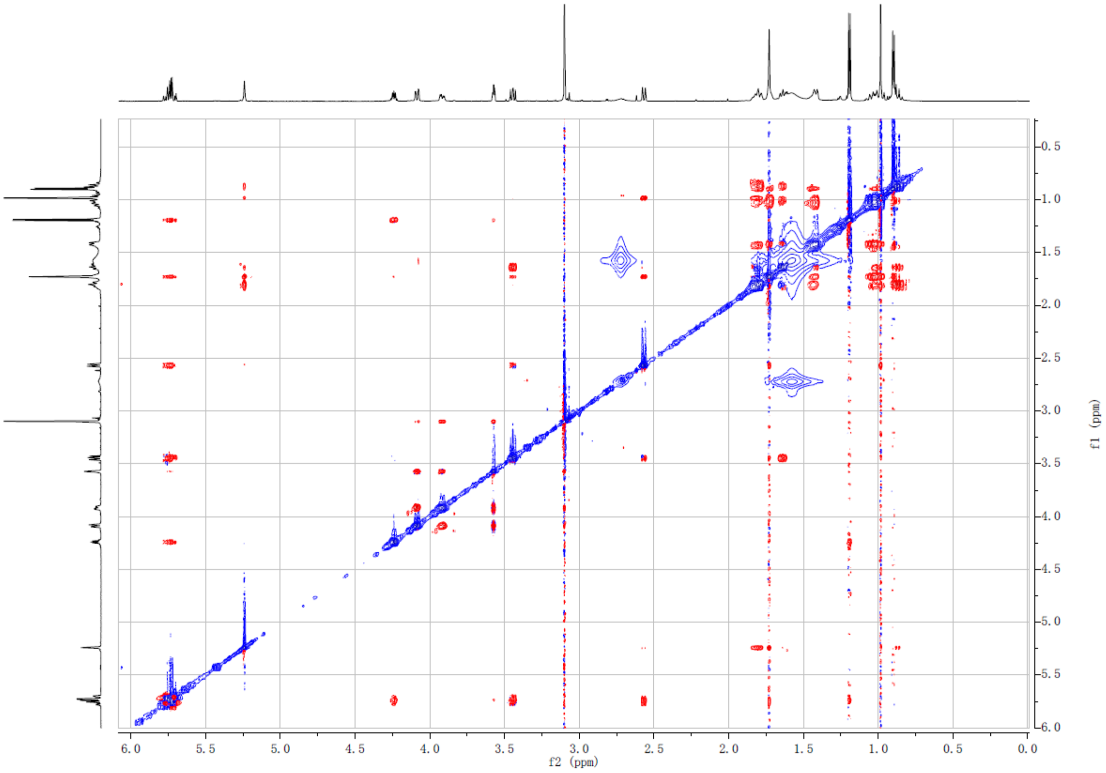

Figure S7. HR-ESIMS spectrum of compound **1**.

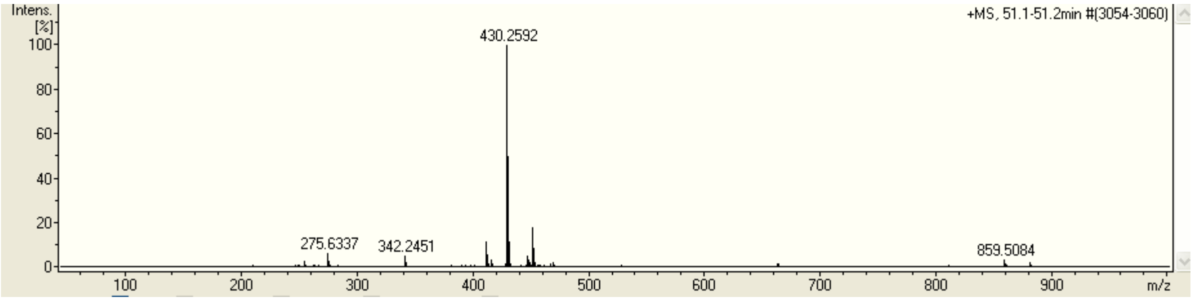

Figure S8. FT-IR spectrum of compound **1**.

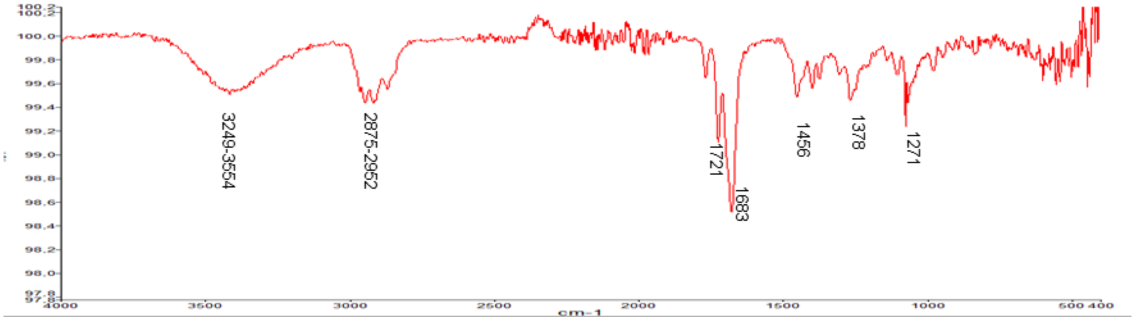

Figure S9. ^{1}H NMR spectrum of compound **2** (600 MHz, $CDCl_3$).

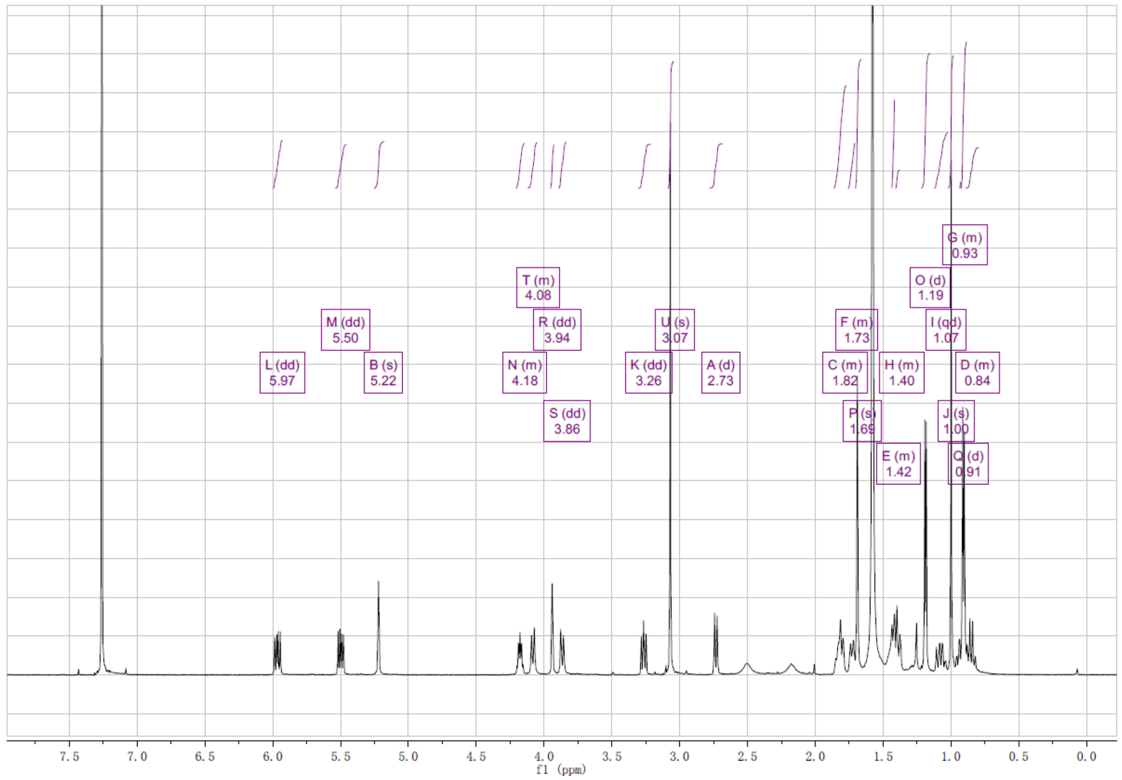

Figure S10. ^{13}C NMR spectrum of compound **2** (150 MHz, $CDCl_3$).

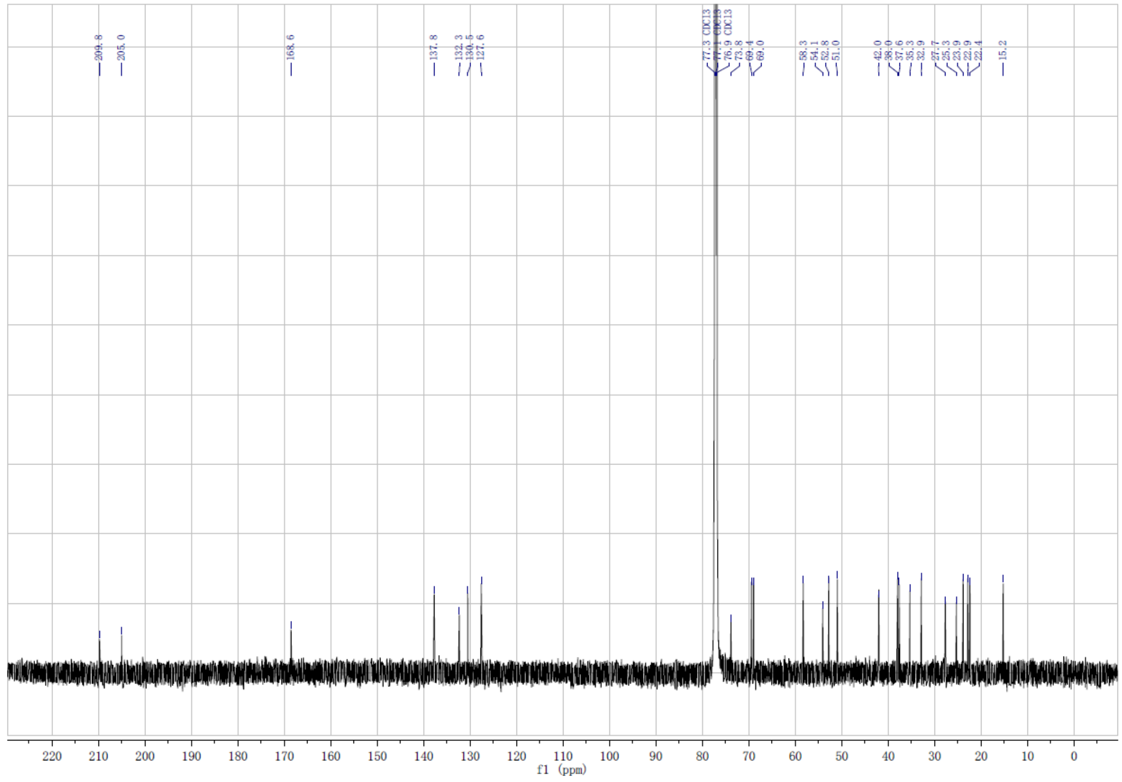

Figure S11. DEPT-HSQC spectrum of compound **2** (600 MHz, $CDCl_3$).

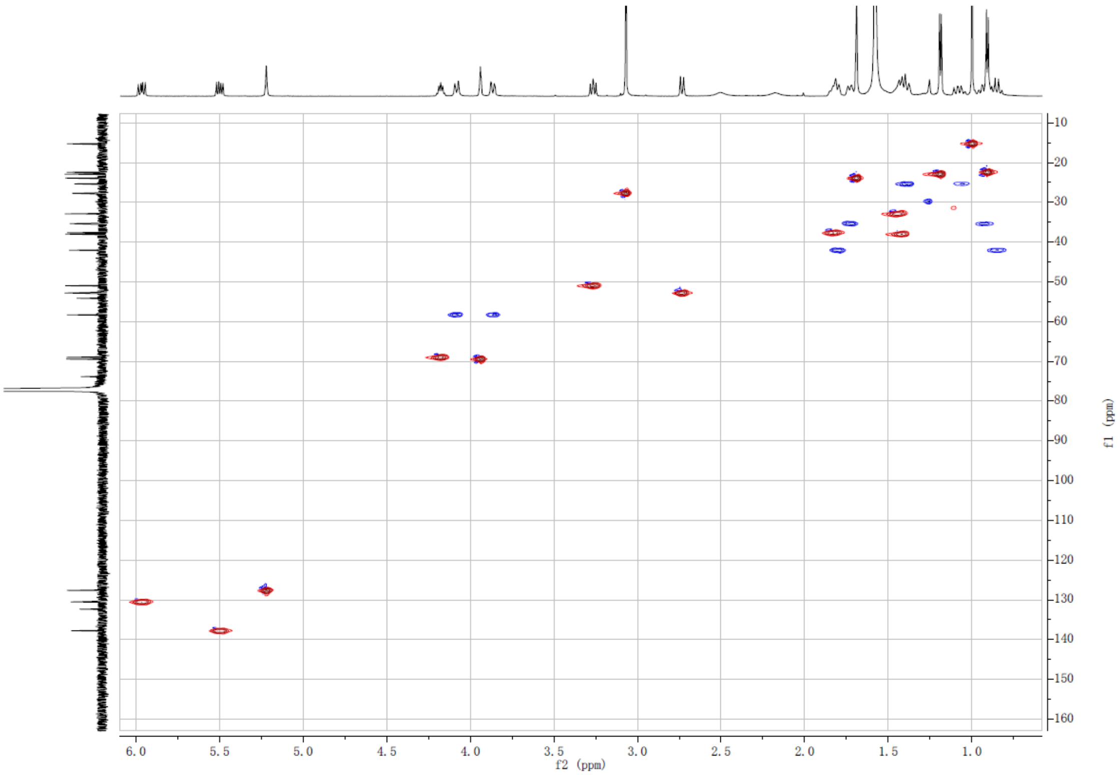

Figure S12. COSY spectrum of compound **2** (600 MHz, $CDCl_3$).

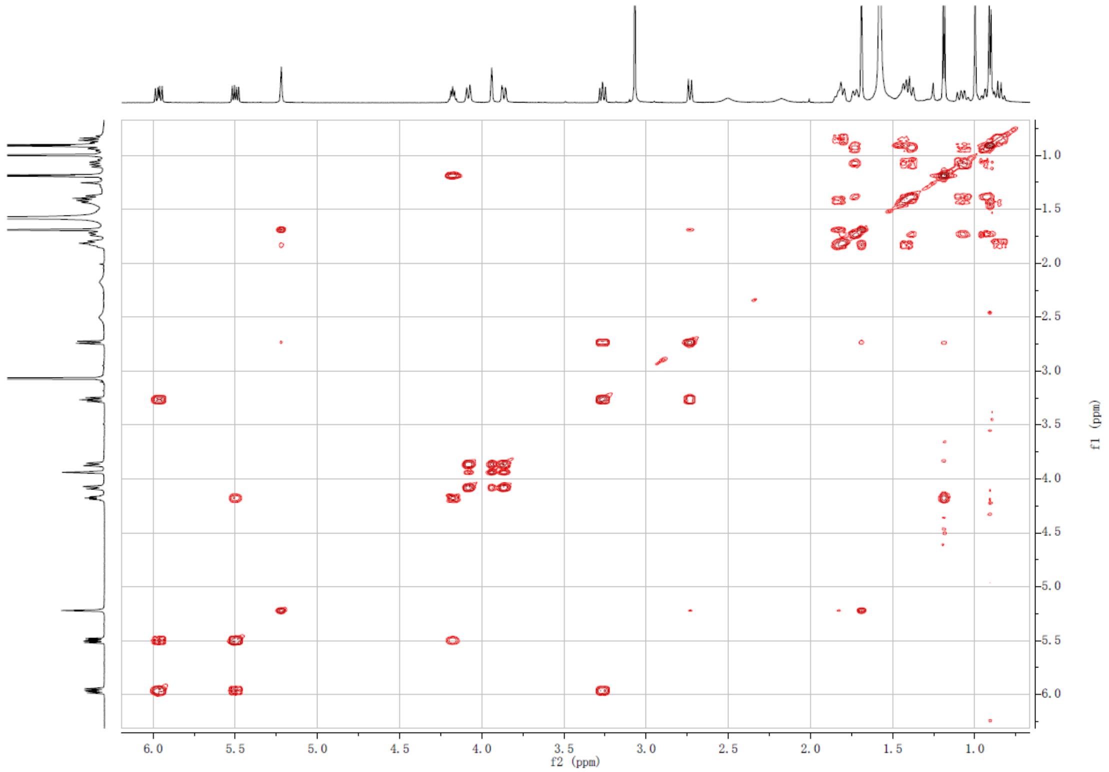

Figure S13. HMBC spectrum of compound **2** (600 MHz, CDCl3).

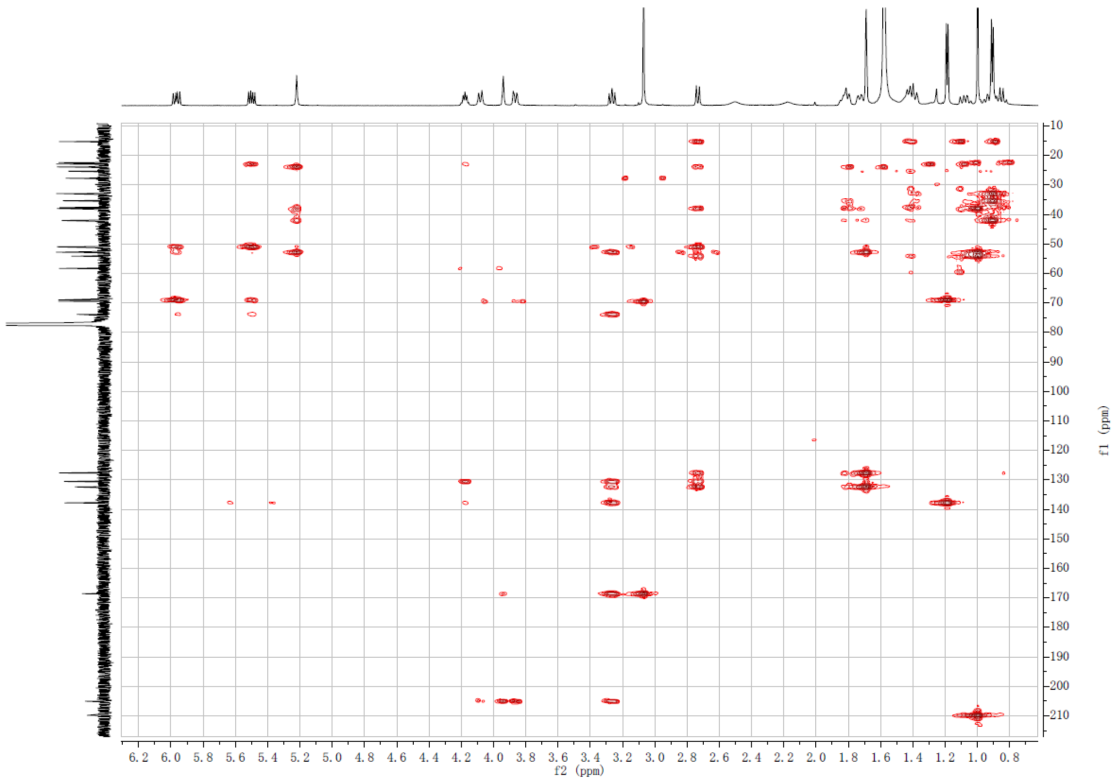

Figure S14. NOESY spectrum of compound **2** (600 MHz, CDCl3).

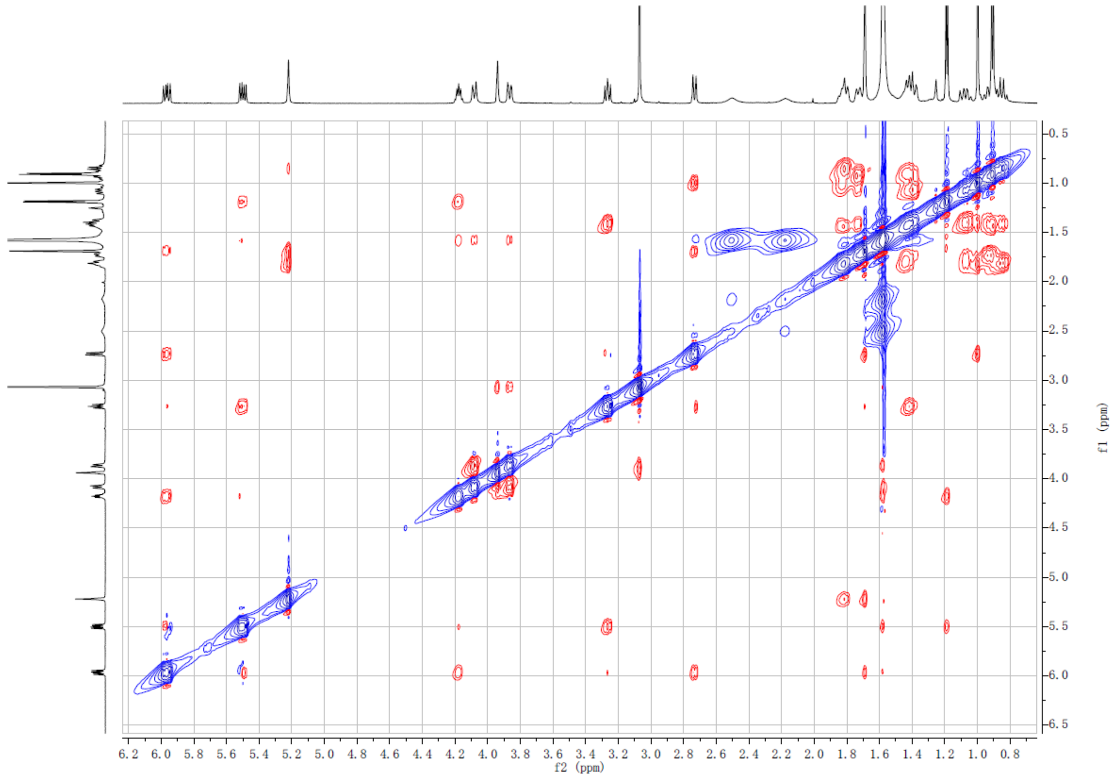

Figure S15. HR-ESIMS spectrum of compound **2**.

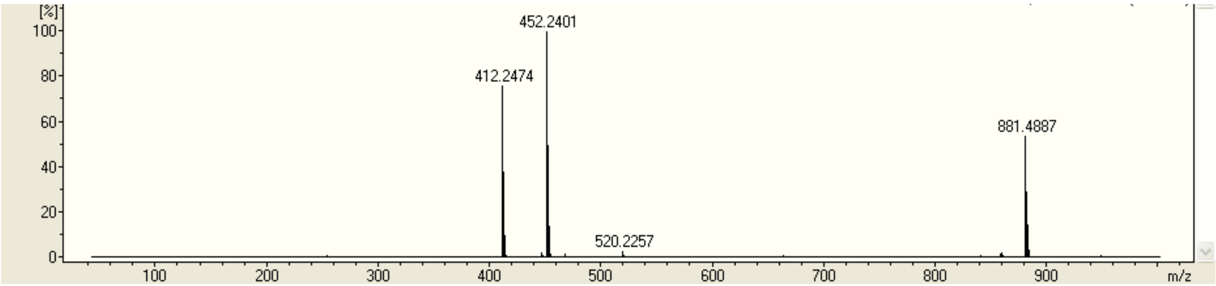

Figure S16. FT-IR spectrum of compound **2**.

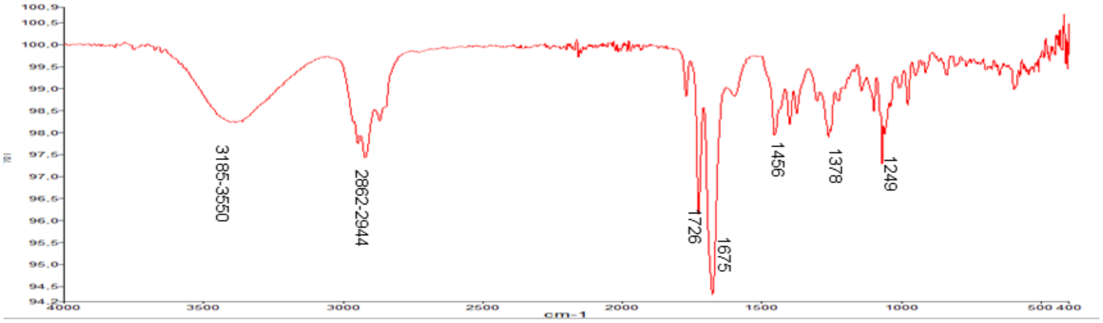

Figure S17. ^{1}H NMR spectrum of compound **3** (600 MHz, $CDCl_3$).

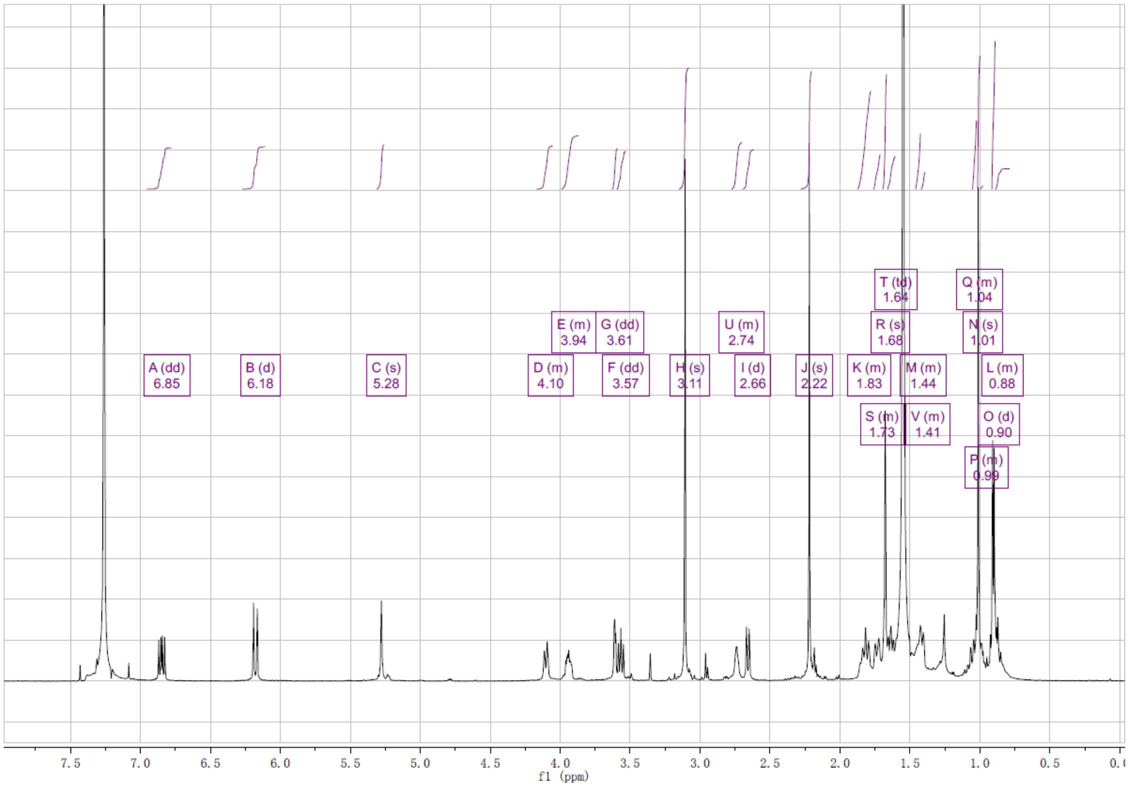

Figure S18. ^{13}C NMR spectrum of compound **3** (150 MHz, $CDCl_3$).

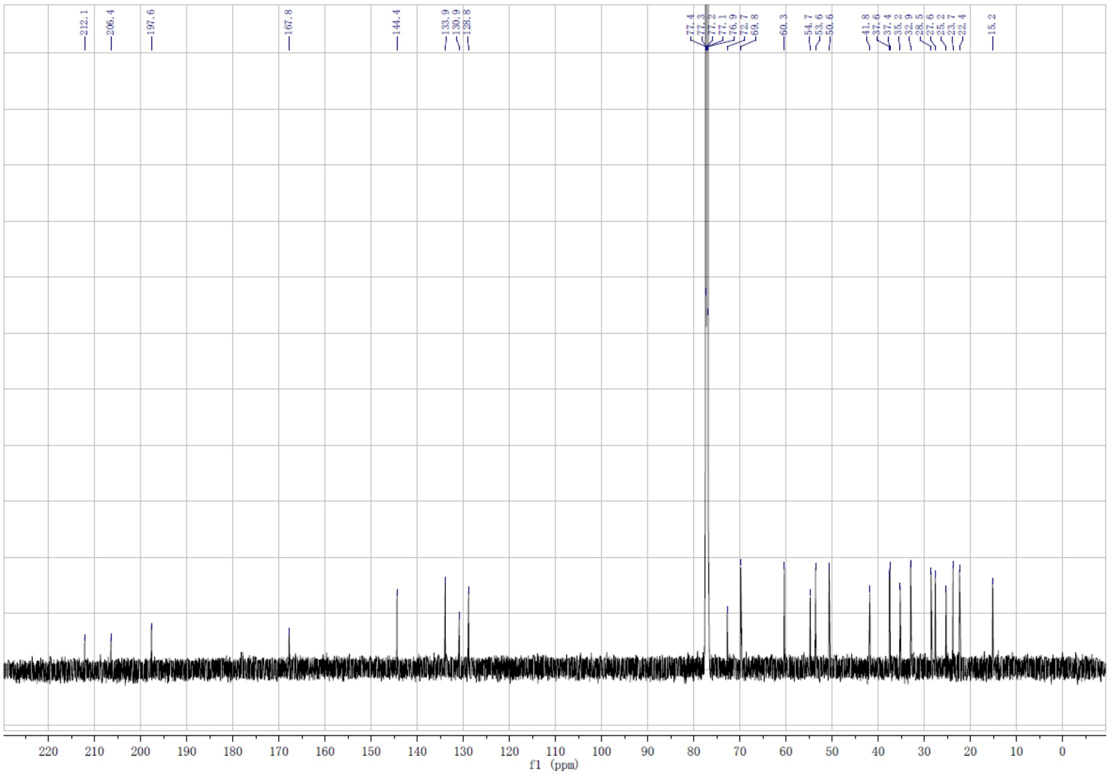

Figure S19. DEPT-HSQC spectrum of compound **3** (600 MHz, CDCl3).

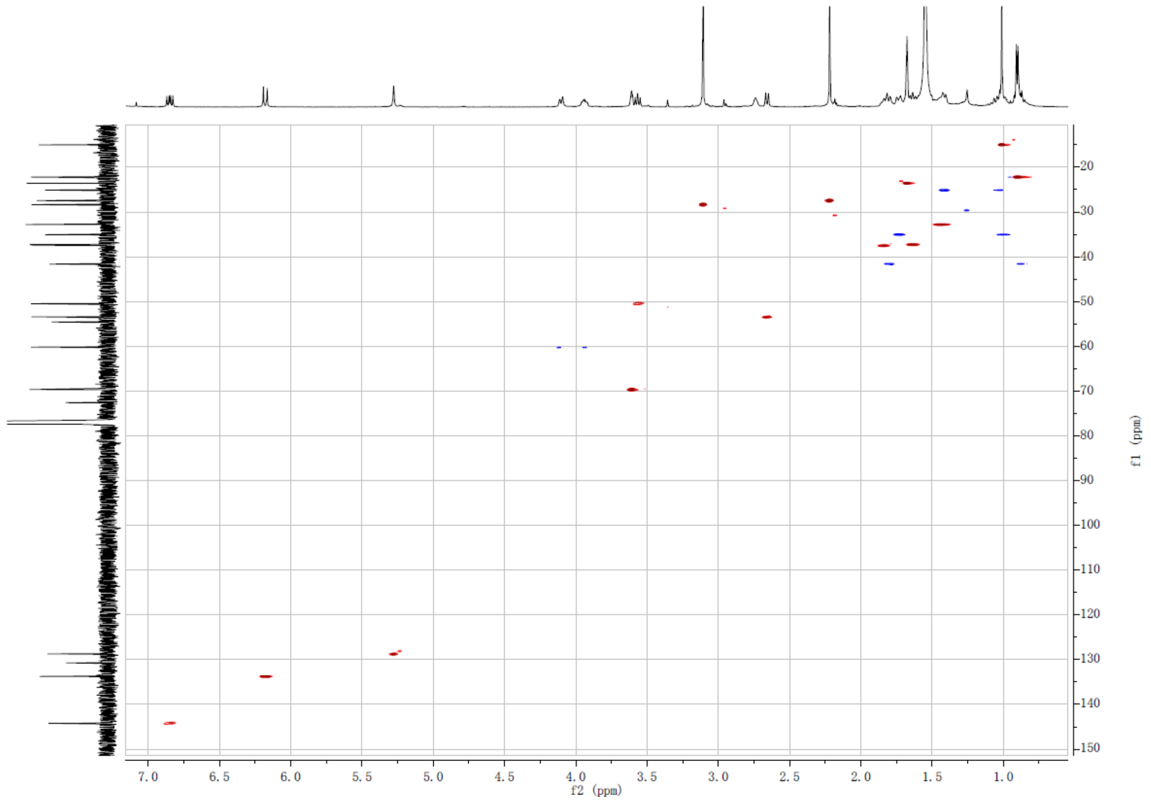

Figure S20. COSY spectrum of compound **3** (600 MHz, CDCl3).

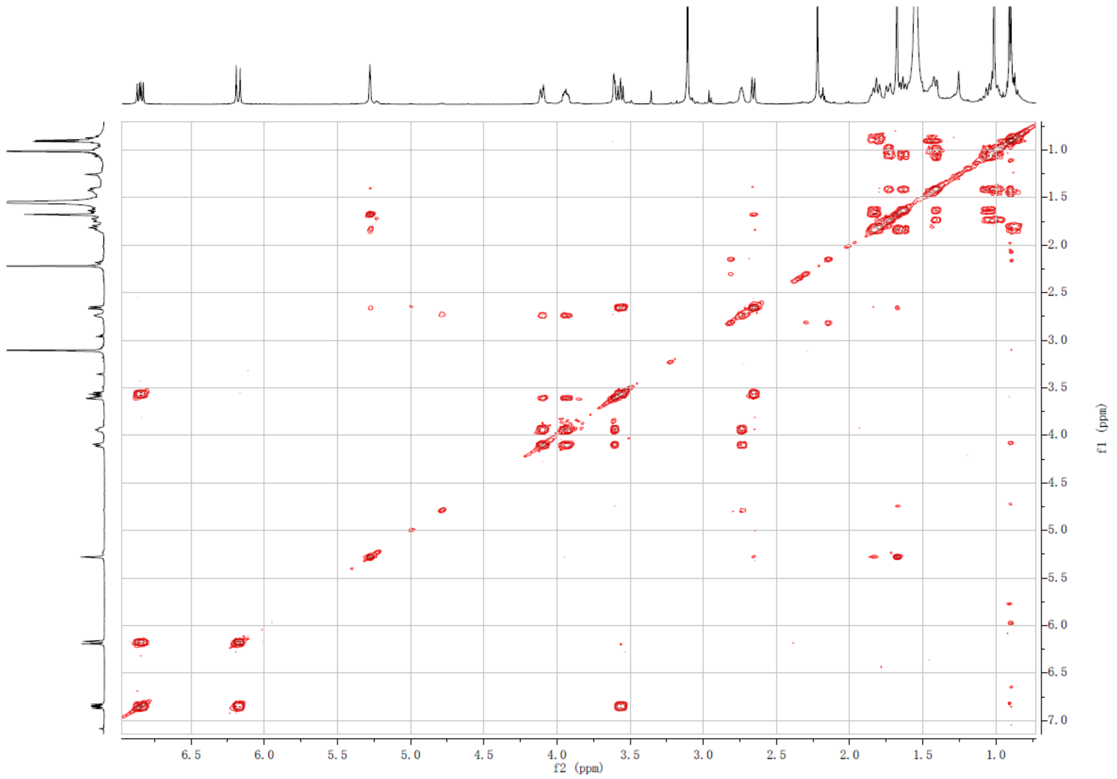

Figure S21. HMBC spectrum of compound **3** (600 MHz, CDCl3).

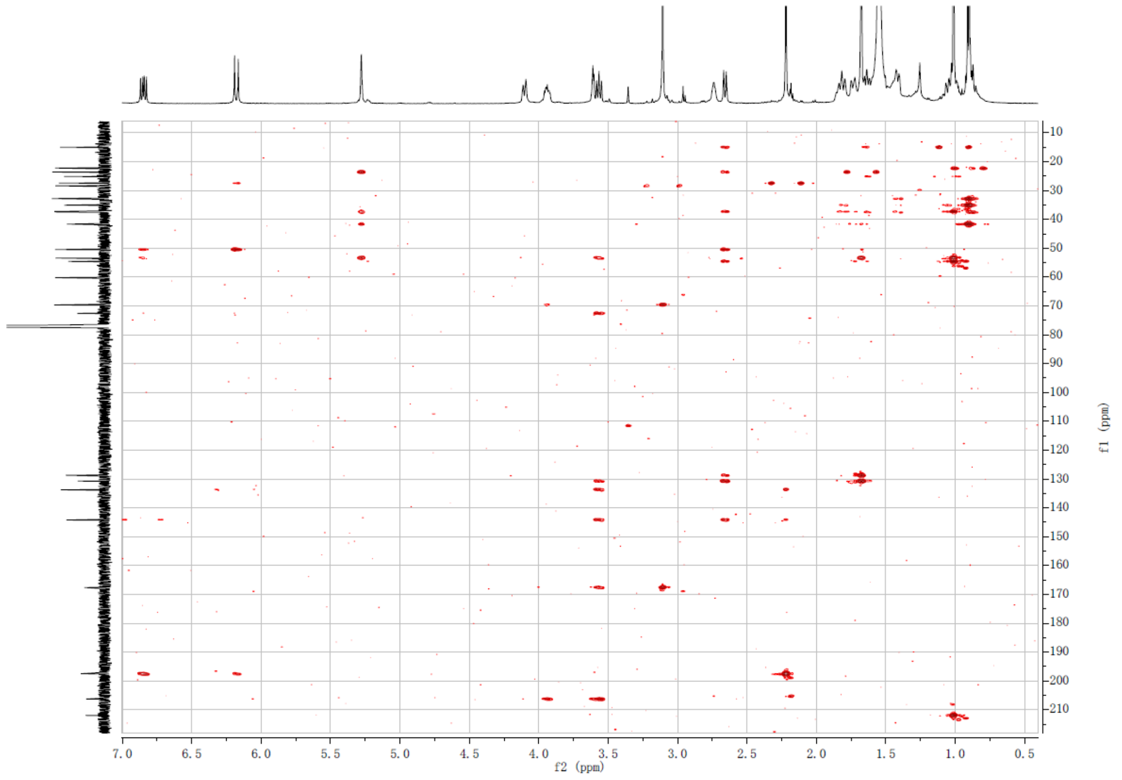

Figure S22. NOESY spectrum of compound **3** (600 MHz, CDCl3).

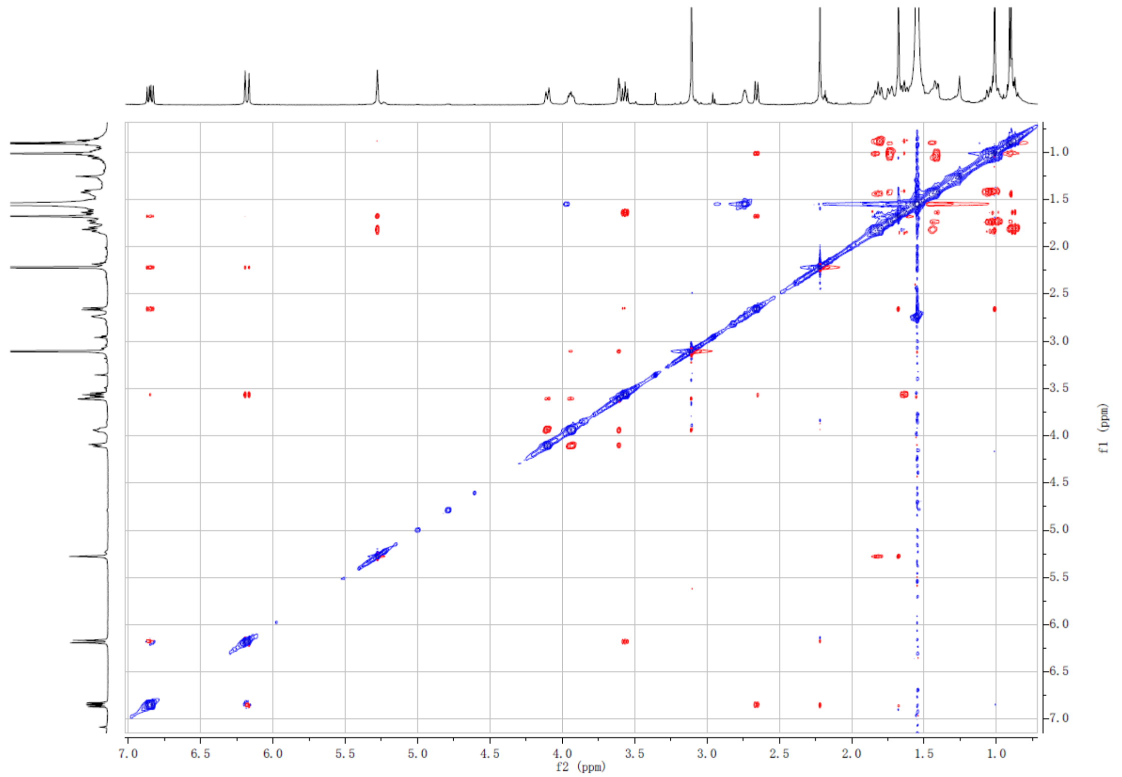

Figure S23. HR-ESIMS spectrum of compound **3**.

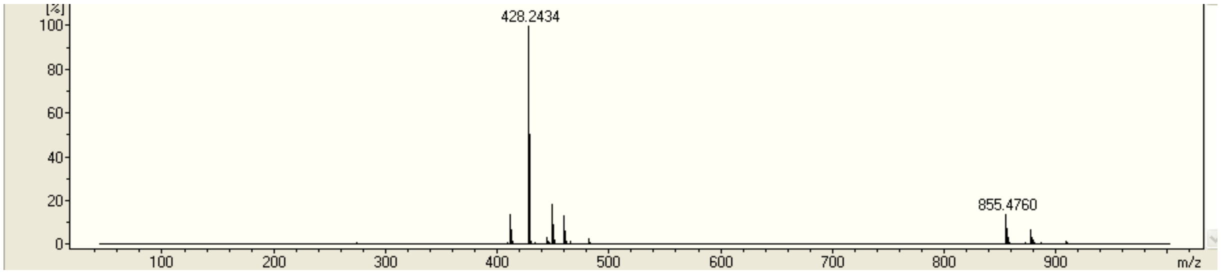

Figure S24. FT-IR spectrum of compound **3**.

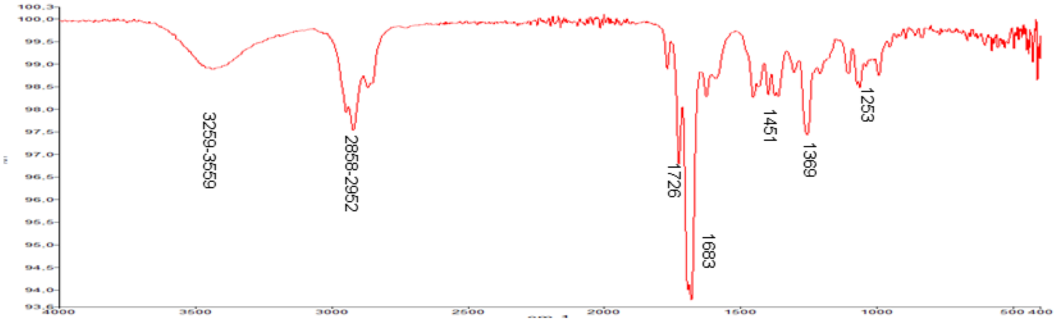

Figure S25. ^{1}H NMR spectrum of 16-(*S*)-MTPA ester **6** (500 MHz, $CDCl_3$).

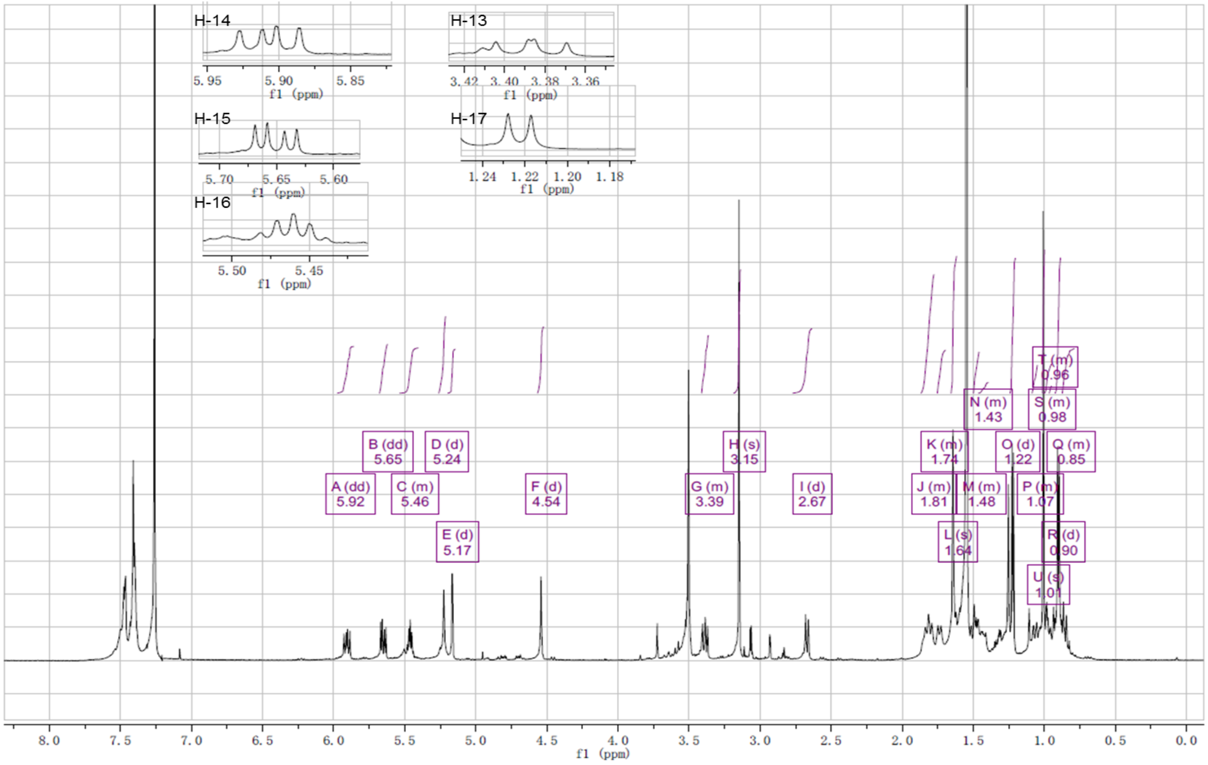

Figure S26. ^{1}H NMR spectrum of 16-(*R*)-MTPA ester **7** (500 MHz, $CDCl_3$).

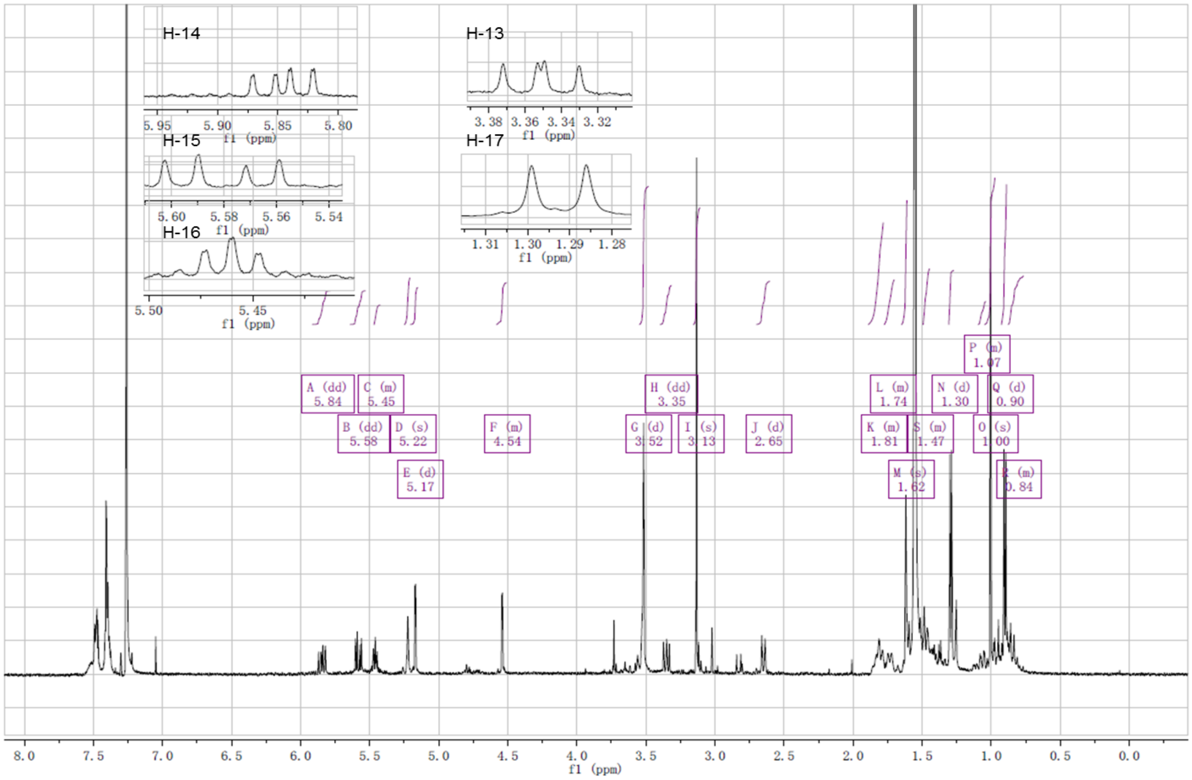

Figure S27. ^{1}H NMR spectrum of 16-(*S*)-MTPA ester **8** (500 MHz, $CDCl_3$).

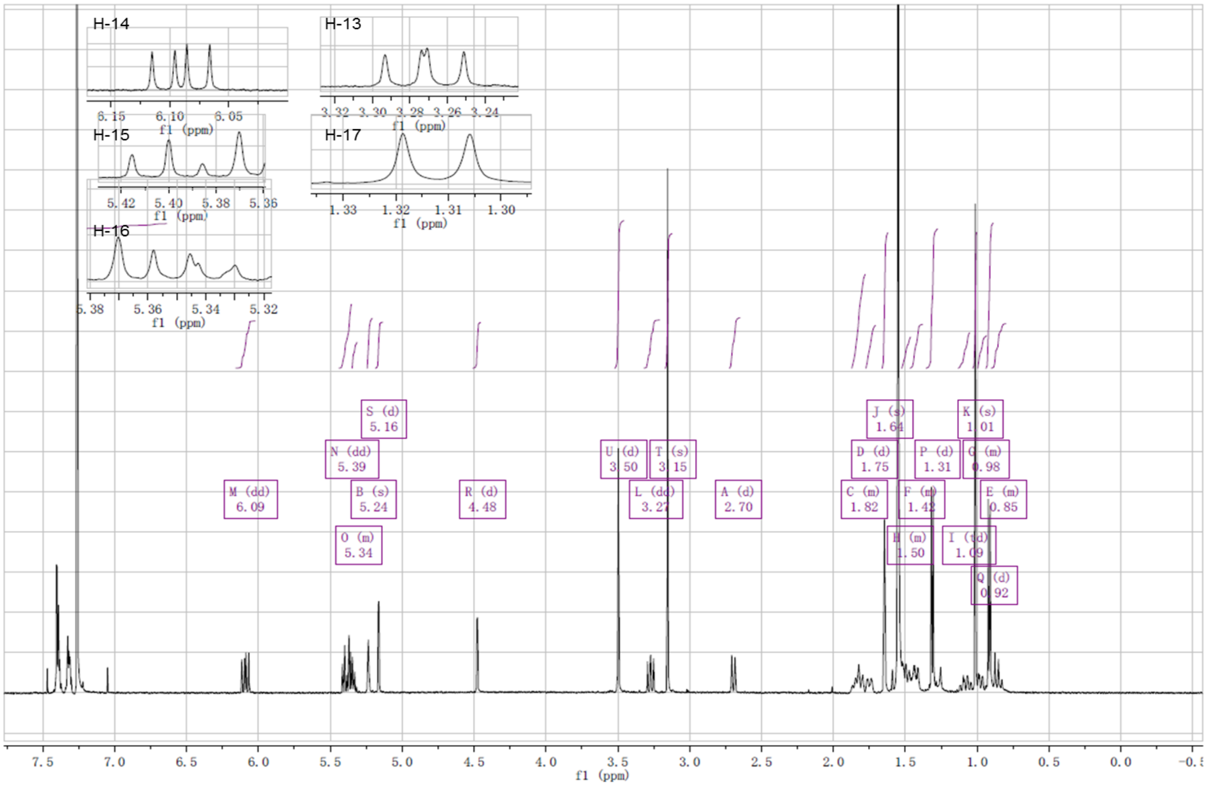

Figure S28. ^{1}H NMR spectrum of 16-(*R*)-MTPA ester **9** (500 MHz, $CDCl_3$).

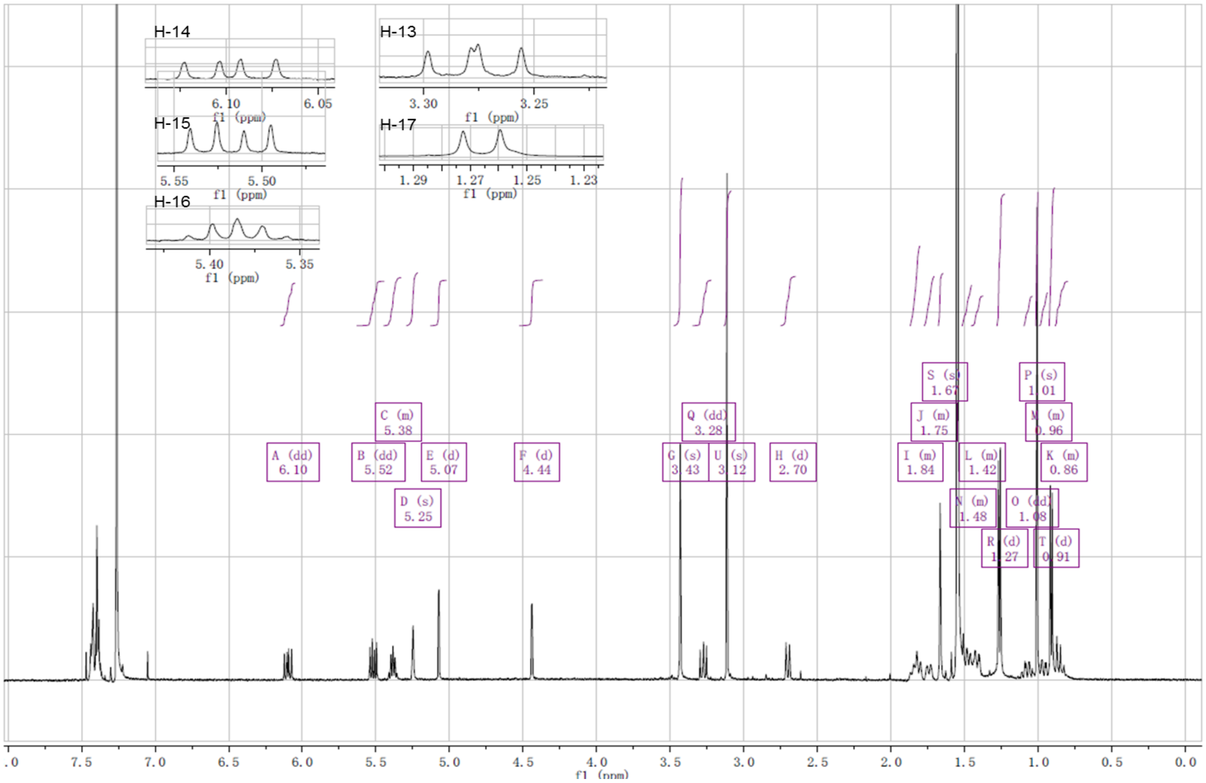

Figure S29. $\Delta\delta(\delta_{S}-\delta_{R})$ values (ppm) obtained from 16-MTPA esters (**6** and **7**) of compound **1**.

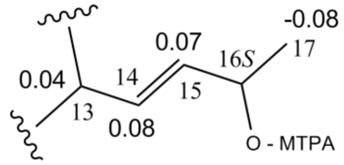

Figure S30. $\Delta\delta(\delta_{S}-\delta_{R})$ values (ppm) obtained from 16-MTPA esters (**8** and **9**) of compound **2**.

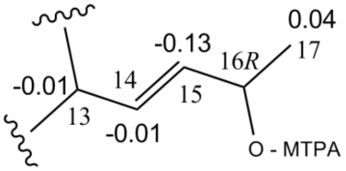

Supplement: Supplementary file 1 [file marinedrugs-18-00047-s001.pdf]
